# Species distribution and in vitro antimicrobial susceptibility of coagulase-negative staphylococci isolated from bovine mastitic milk

**DOI:** 10.1186/s13028-016-0193-8

**Published:** 2016-02-06

**Authors:** Suvi Taponen, Suvi Nykäsenoja, Tarja Pohjanvirta, Anna Pitkälä, Satu Pyörälä

**Affiliations:** 1 Department of Production Animal Medicine, Faculty of Veterinary Medicine, University of Helsinki, Paroninkuja 20, 04920 Saarentaus, Finland; 2 Finnish Food Safety Authority Evira, Mustialankatu 3, 00790 Helsinki, Finland

**Keywords:** Coagulase-negative staphylococci, CoNS species, Antimicrobial resistance, Bovine, Cow, MIC, *Staphylococcus epidermidis*, *Staphylococcus chromogenes*, *Staphylococcus haemolyticus*, *Staphylococcus simulans*

## Abstract

**Background:**

Coagulase-negative staphylococci (CoNS) are the most common bovine mastitis causing bacteria in many countries. It is known that resistance for antimicrobials is in general more common in CoNS than in *Staphylococcus aureus* but little is known about the antimicrobial resistance of specific CoNS species. In this study, 400 CoNS isolates from bovine mastitic milk samples were identified to species level using ribotyping and MALDI-TOF MS, and their antimicrobial susceptibility was determined using a commercially available microdilution system. The results were interpreted according to the epidemiological cut-off values by the European Committee on Antimicrobial Susceptibility testing.

**Results:**

The most common CoNS species were *S. simulans, S. epidermidis, S. chromogenes* and *S. haemolyticus.* Penicillin resistance was the most common type of antimicrobial resistance. *Staphylococcus epidermidis* was the most resistant among the four major species. Almost one-third of our *S. epidermidis* isolates were resistant to >2 antimicrobials and close to 7 % were multidrug resistant. The majority of *S. epidermidis* isolates were resistant to benzylpenicillin. On the contrary, only few *S. simulans* isolates were penicillin-resistant. Phenotypic oxacillin resistance was found in all four main species, and 34 % of the isolates were oxacillin resistant. However, only 21 isolates (5 %) were positive for the *mec*A gene. Of these, 20 were *S. epidermidis* and one *S. sciuri*. *mec*C positive isolates were not found.

**Conclusion:**

*Staphylococcus epidermidis* differed from the three other major CoNS species as resistance to the tested antimicrobials was common, several isolates were multidrug resistant, and 19 % of the isolates carried the *mec*A gene encoding methicillin resistance.

**Electronic supplementary material:**

The online version of this article (doi:10.1186/s13028-016-0193-8) contains supplementary material, which is available to authorized users.

## Background

Prevalence of mastitis in dairy cows and distribution of mastitis-causing bacteria has regularly been monitored in Finland [1, 2]. These surveys have also reported antimicrobial in vitro susceptibility of different bacterial species, including coagulase-negative staphylococci (CoNS). Coagulase-negative staphylococci have become the most common mastitis causing agents in many countries [3]. They mostly cause subclinical mastitis but have also been isolated from clinical mastitis [3, 4]. It is known that resistance for antimicrobials is in general more common in CoNS than in *S. aureus* [4]. The most common resistance among bovine CoNS is production of β-lactamase which confers resistance to benzylpenicillin and aminopenicillins, but also resistance towards aminoglycosides, tetracyclines, and macrolides has been reported [2, 5, 6]. Methicillin-resistant CoNS have been isolated from bovine mastitis which is of special concern because of the risk of spreading the *mec* genes [7, 8]. Furthermore, emergence of resistance among CoNS is a concern because resistance determinants may be transferred between staphylococcal species and form a risk for public health [9, 10].

Phenotypic identification methods for bovine CoNS have proven to be unsatisfactory [11–13]. Molecular methods have become available for identification of CoNS to species level, which has made species determination more reliable. Reliable genotypic identification has enabled studying frequency of different CoNS species and species-specific antimicrobial susceptibility. Reports on mastitis causing CoNS species and their antimicrobial susceptibility have since been published by some authors [5, 6, 14]. Unfortunately, only few studies have used epidemiological cut-off values (ECOFF) of the European Committee on Antimicrobial Susceptibility testing to determine proportions of resistant isolates [15], which has made comparisons difficult. Studies on genetic mechanisms for resistance of bovine CoNS species have also been published, with different panels of resistance genes [5, 16, 17]. In Finland, antimicrobial susceptibility of CoNS has been reported for bovine CoNS as a group only [2, 18]. It is likely that in the future CoNS will no more be considered as one homogenous group, but species-specific approaches become possible in mastitis control [3]. Knowledge on the antimicrobial susceptibility of different CoNS species is then also necessary.

The aim of this study was to explore the distribution of CoNS species isolated from mastitic milk samples in Finland and to determine the antimicrobial susceptibility of different CoNS species.

## Methods

The material consists of CoNS isolates from two studies, Pitkälä et al. [2] (dataset 1) and Finnish veterinary antimicrobial resistance monitoring program 2010–2012 [18] (dataset 2). The number of CoNS isolates was 312 and 88 in dataset 1 and 2, respectively.

The first study (dataset 1) was a nationwide prevalence study carried out in 2001, in which milk samples were collected from all four quarters of all lactating cows in herds randomly allocated into the study. Conventional microbiological methods were used to identify bacteria isolated from the milk samples [2, 19]. The milk samples were classified as mastitic when the milk somatic cell count, measured with an electric counter (Fossomatic Milk Analysis, Foss Electric, Hillerød, Denmark), exceeded 300 000 cells/ml. Of the total of 2103 CoNS isolated in that study, 335 were randomly chosen for in vitro antimicrobial susceptibility testing [2]. Of these isolates, 318 were successfully identified to species level in the present study. Six isolates appeared to belong to *S. aureus* species and were withdrawn from this study. In the second study (dataset 2) the samples were routine mastitis samples (based on elevated milk somatic cell count and/or clinical signs of mastitis) submitted during 2012 to the laboratory of Valio Ltd by veterinarians and dairy farmers. Bacteriological etiology of mastitis was determined by a real-time PCR assay (Thermo Scientific PathoProof™ Mastitis Complete-12 Kit, Thermo Fisher Scientific Ltd.,Vantaa, Finland). During the study, the preservative was left out from the milk tubes. Samples positive for CoNS in the PCR test were selected for culture. From the cultured pure samples the first cultured 88 CoNS isolates, but only one isolate per herd, were selected for determination of antimicrobial susceptibility. *Staphylococcus* species identification was performed during the present study.

The CoNS isolates from both datasets were identified to species level with the 16S and 23S rRNA gene restriction fragment length polymorphism method (ribotyping) as described previously [20]. The CoNS species were determined by comparison in a numerical similarity analysis of the ribotype patterns with a ribotype library using BioNumerics 5.1 software package (Applied Maths, St.-Martens-Latem, Belgium). For some isolates ribotyping failed, and they were later analyzed with MALDI-TOF MS (Microflex LT, Bruker Daltonic Gmbh, Bremen, Germany). The correctness of species identification based on ribotyping was confirmed by analyzing a representative sample of all different ribotype patterns by MALDI-TOF MS [21]. The agreement between the methods was excellent.

Antimicrobial susceptibility of CoNS isolates from both datasets was determined in the previous studies [2, 18] using a commercially available microdilution system (VetMIC™; SVA, Uppsala, Sweden). Minimum inhibitory concentrations (MIC) in both datasets were determined for penicillin, cephalothin, oxacillin, erythromycin, chloramphenicol, clindamycin, tetracycline, gentamicin, neomycin, streptomycin, and trimethoprim/sulfamethoxazole. In addition, MICs for virginiamycin, vancomycin and avilamycin in dataset 1, and for fusidic acid, kanamycin, ciprofloxacin, trimethoprim, florfenicol, and cefoxitin in dataset 2, were determined. Results from the susceptibility testing were interpreted according to the epidemiological cut-off values (ECOFFs) by the European Committee on Antimicrobial Susceptibility testing (EUCAST) [22] as non-wild type (from now on referred as resistant) or wild type (sensitive). If a specific ECOFF was not available for the specific species or for CoNS as a group, ECOFF of *Staphylococcus aureus* was used. Production of beta-lactamase was tested using nitrocefin discs (dataset 1: AB Biodisk, Solna Sweden; dataset 2: Becton–Dickinson, NJ, USA). The isolates with MIC values for oxacillin >1 mg/l were tested for presence of the *mec*A gene in dataset 1 and for presence of the *mec*A and *mec*C genes in dataset 2, using PCR and primers reported previously [23, 24].

## Results

### Distribution of CoNS species

The numbers and proportions of isolates of different CoNS species are shown in Table 1. In dataset 1, a total of 14 staphylococcal species were identified. The most common CoNS species were *S. simulans* (25.0 %), *S. epidermidis* (25.0 %), *S. chromogenes* (15.4 %), *S. haemolyticus* (11.9 %), and *S. warneri* (10.3 %). Three isolates could not be identified by ribotyping or by MALDI-TOF MS and were grouped as *Staphylococcus* sp. In dataset 2, similarly as in the dataset 1, *S. simulans* (34.1 %) and *S. epidermidis* (30.7 %) were the most common species. *Staphylococcus chromogenes* was the third most common species (6.8 %). The proportion of both *S. haemolyticus* and *S. cohnii* isolates was 5.7 %. Three isolates could not be identified by ribotyping or MALDI-TOF MS and were grouped as *Staphylococcus* sp. The unidentified isolates may represent a new *Staphylococcus* species or one of the few CoNS species, like *S. devriesei*, which were not included in the ribotype and MALDI-TOF MS comparison databases at the time of the analyses. The four most common CoNS species represented 77.3 % of all 400 isolates.

**Table 1:** Distribution of coagulase-negative *Staphylococcus* species isolated in bovine milk samples in 2001 (dataset 1) and 2012 (dataset 2)

CNS species	2001		2012		In total	
	n	%	n	%	n	%
*S. agnetis*	7	2.2	3	3.4	10	2.5
*S. capitis*	1	0.3	1	1.1	2	0.5
*S. chromogenes*	48	15.4	6	6.8	54	13.5
*S. cohnii*	7	2.2	5	5.7	12	3.0
*S. epidermidis*	78	25.0	27	30.7	105	26.3
*S. equorum*	3	1.0	0	0	3	0.8
*S. haemolyticus*	37	11.9	5	5.7	42	10.5
*S. hyicus*	4	1.3	1	1.1	5	1.3
*S. kloosii*	0	0	3	3.4	3	0.75
*S. pasteuri*	2	0.6	0	0	2	0.5
*S. saprophyticus*	2	0.6	1	1.1	3	0.8
*S. sciuri*	2	0.6	1	1.1	3	0.8
*S. simulans*	78	25.0	30	34.1	108	27.0
*S. warneri*	32	10.3	1	1.1	33	8.3
*S. xylosus*	8	2.6	2	2.3	10	2.5
*Staphylococcus* sp.	3	1.0	2	2.3	5	1.3
In total	312		88		400	

The material consisted of CoNS isolates from two studies, Pitkälä et al. [2] (dataset 1) and Finnish veterinary antimicrobial resistance monitoring program 2010–2012 [18] (dataset 2). In dataset 1 all quarters of cows were sampled in a mastitis survey, in dataset 2 samples originated from quarters with mastitis

### In vitro antimicrobial susceptibility

The four major CoNS species differed in their in vitro antimicrobial susceptibility. Among them, antimicrobial resistance was most common in *S. epidermidis* (Tables 2 and 3). MIC distributions of the four major CoNS species for the tested antimicrobials in 2001 and 2012 are shown in Tables 2 and 3. The MIC results of all 400 isolates by species are shown in the Additional file 1: Table S1 and as a CoNS group in the Additional file 2: Table S2.

**Table 2:** In vitro susceptibility to 14 antimicrobials of *S. chromogenes*, *S. epidermidis*, *S. haemolyticus* and *S. simulans* isolated in bovine milk samples from dataset 1 (2001)

Antimicrobial	Organism	N	N (%) > ECOFF	MIC mg/l, % of isolates													ECOFF^a^
Benzylpenicillin				≤*0.06*	*0.12*	*0.25*	*0.5*	*1*	*2*	*4*	*8*	≥*16*					*0.125* ^b^
	*S. chromogenes*	48	14 (29.2)	68.8	2.1	4.2	6.3	12.5	6.3								
	*S. epidermidis*	78	58 (74.4)	23.1	2.6	6.4	9.0	12.8	15.4	9.0	11.5	10.3					
	*S. haemolyticus*	37	24 (64.9)	32.4	2.7	16.2	27.0	13.5	5.4	2.7							
	*S. simulans*	78	3 (3.8)	93.6	2.6	2.6			1.3								
Oxacillin							≤*0.5*	*1*	*2*	*4*	≥*8*						*1.0*
	*S. chromogenes*	48	19 (39.6)				20.8	39.6	37.5	2.1							
	*S. epidermidis*	78	27 (34.6)				38.5	26.9	15.4	6.4	12.8						
	*S. haemolyticus*	37	10 (27.0)				32.4	40.5	24.3	2.7							
	*S. simulans*	78	15 (19.2)				38.5	42.3	19.2								
Cephalothin					≤*0.12*	*0.25*	*0.5*	*1*	*2*	*4*	≥*8*						*1.0* ^b^
	*S. chromogenes*	48			22.9	70.8	6.3										
	*S. epidermidis*	78			21.8	55.1	9.0	14.1									
	*S. haemolyticus*	37			10.8	67.6	21.6										
	*S. simulans*	78			6.4	56.4	37.2										
Streptomycin									≤*2*	*4*	*8*	*16*	*32*	*64*	*128*	≥*256*	*16.0* ^b^
	*S. chromogenes*	48							81.3	14.6	4.2						
	*S. epidermidis*	78	17 (21.8)						71.8	2.6	2.6	1.3	2.6	5.1	6.4	7.7	
	*S. haemolyticus*	37							97.3	2.7							
	*S. simulans*	78	1 (1.3)						68.2	19.2	5.1	5.1			1.3		
Neomycin								≤*2*	*2*	*4*	*8*	*16*	*32*	≥*64*			*1.0* ^b^
	*S. chromogenes*	48						97.9	2.1								
	*S. epidermidis*	78						89.7		1.3	3.8	3.8	1.3				
	*S. haemolyticus*	37						100.0									
	*S. simulans*	78						100.0									
Gentamicin						≤*0.25*	*0.5*	*1*	*2*	*4*	*8*	≥*16*					*0.5*
	*S. chromogenes*	48				95.8	4.2										
	*S. epidermidis*	78				100.0											
	*S. haemolyticus*	37				100.0											
	*S. simulans*	78	2 (2.6)			88.5	9.0	2.6									
Clindamycin								≤*1*	*2*	*4*	≥*8*						*0.25*
	*S. chromogenes*	48						100.0									
	*S. epidermidis*	78						100.0									
	*S. haemolyticus*	37						97.3		2.7							
	*S. simulans*	78						100.0									
Erythromycin							≤*0.5*	*1*	*2*	*4*	≥*8*						*1.0*
	*S. chromogenes*	48	2 (4.2)				95.8				4.2						
	*S. epidermidis*	78	11 (14.1)				84.6	1.3			14.1						
	*S. haemolyticus*	37	1 (2.7)				94.6	2.7			2.7						
	*S. simulans*	78					100.0										
Chloramphenicol									≤*2*	*4*	*8*	*16*	≥*32*				*16.0*
	*S. chromogenes*	48							4.2	77.1	18.8						
	*S. epidermidis*	78	2 (2.6)						1.3	87.2	1.3	7.7	2.6				
	*S. haemolyticus*	37							10.8	86.5	2.7						
	*S. simulans*	78							3.8	47.4	46.2	2.6					
Tetracycline							≤*0.5*	*1*	*2*	*4*	*8*	*16*	*32*	*64*	≥*128*		*1.0*
	*S. chromogenes*	48	3 (6.2)				87.5	6.3						2.1	4.2		
	*S. epidermidis*	78	36 (46.2)				47.4	6.4	7.7	20.5	2.6		1.3	3.8	10.3		
	*S. haemolyticus*	37					75.7	24.3									
	*S. simulans*	78	4 (5.1)				21.8	73.1	2.6	1.3				1.3			
Trimetoprim-sulfamethoxazole						≤*0.25/4.25*	*0.5/9.5*	*1/19*	*2/38*	*4/76*	*8/152*	≥*16/304*					*0.5/9.5*
	*S. chromogenes*	48	5 (10.4)			75.0	14.6	4.2		2.1	2.1	2.1					
	*S. epidermidis*	78	7 (9.0)			73.1	17.9		5.1	2.6		1.3					
	*S. haemolyticus*	37				89.2	10.8										
	*S. simulans*	78	6 (7.7)			29.5	62.8	6.1	2.6								
Vancomycin								≤*1*	*2*	*4*	*8*	*16*	*32*	≥*64*			*4.0*
	*S. chromogenes*	48						83.3	16.7								
	*S. epidermidis*	78						26.9	73.1								
	*S. haemolyticus*	37						81.1	18.9								
	*S. simulans*	78	1 (1.3)					91.0	6.4	1.3			1.3				
Virginiamycin							≤*0.5*	*1*	*2*	*4*	≥*8*						*1.0* ^c^
	*S. chromogenes*	48	2 (4.2)				25.0	70.8	4.2								
	*S. epidermidis*	78	1 (1.3)				65.4	33.3	1.3								
	*S. haemolyticus*	37	1 (2.7)				37.8	59.5	2.7								
	*S. simulans*	78	2 (2.6)				15.4	82.1	1.3	1.3							
Avilamycin							≤*0.5*	*1*	*2*	*4*	*8*	*16*	*32*	≥*64*			*ND*
	*S. chromogenes*	48							27.1	54.2	18.8						
	*S. epidermidis*	78								29.5	65.4	5.1					
	*S. haemolyticus*	37							16.2	59.5	21.6	2.7					
	*S. simulans*	78						1.3	1.3	11.5	37.2	33.3	15.4				

In this dataset, all quarters of cows were sampled in a mastitis survey [2]

*ND* not determined

^a^Current (March 2015) EUCAST epidemiological cut-off (ECOFF) values (mg/l) for CNS were used to define resistant isolates. If ECOFF for CNS was not available, the value for *S. aureus* was used. For virginiamycin, ECOFF for *S. intermedius* was used

^b^ECOFF for *S. aureus*

^c^ECOFF for *S. intermedius*

**Table 3:** In vitro susceptibility to 17 antimicrobials of *S. chromogenes*, *S. epidermidis*, *S. haemolyticus* and *S. simulans* isolated in bovine milk samples from dataset 2 (2012)

Antimicrobial	Organism	N	N (%) > ECOFF	MIC mg/l, % of isolates													ECOFF^a^
Benzylpenicillin				≤*0.03*	*0.06*	*0.12*	*0.25*	*0.5*	*1*	*2*	*4*	≥*8*					*0.125* ^b^
	*S. chromogenes*	6	1 (16.7)	33.3	50.0				16.7								
	*S. epidermidis*	27	20 (74.1)	18.5	3.7	3.7	11.1	7.4	18.5	7.4	22.2	7.4					
	*S. haemolyticus*	5	2 (40.0)	20.0	40.0				40.0								
	*S. simulans*	30	2 (6.6)	63.3	30.0				3.3	3.3							
Oxacillin						≤*0.12*	*0.25*	*0.5*	*1*	*2*	*4*	*8*	*16*	≥*32*			*1.0*
	*S. chromogenes*	6				16.7	16.7	66.7									
	*S. epidermidis*	27	4 (14.8)				3.7	66.7	14.8		3.7	3.7		7.4			
	*S. haemolyticus*	5						80.0	20.0								
	*S. simulans*	30	2 (6.6)				6.7	36.7	50.0	3.3	3.3						
Cephalothin					≤*0.06*	*0.12*	*0.25*	*0.5*	*1*	*2*	*4*	≥*8*					*1.0* ^b^
	*S. chromogenes*	6				50.0	50.0										
	*S. epidermidis*	27			3.7	40.7	37.0	11.1	7.4								
	*S. haemolyticus*	5				20.0	40.0	40.0									
	*S. simulans*	30				6.7	70.0	23.3									
Streptomycin											≤*4*	*8*	*16*	*32*	≥*64*		*16.0* ^b^
	*S. chromogenes*	6									83.3		16.7				
	*S. epidermidis*	27	2 (7.4)								81.5	11.1		3.7	3.7		
	*S. haemolyticus*	5									100.0						
	*S. simulans*	30	1 (3.3)								73.3	20.0	3.3		3.3		
Neomycin											≤*4*	*8*	*16*	*32*	≥*64*		*1.0* ^b^
	*S. chromogenes*	6									100.0						
	*S. epidermidis*	27									96.3			3.7			
	*S. haemolyticus*	5									100.0						
	*S. simulans*	30									100.0						
Gentamicin								≤*0.5*	*1*	*2*	*4*	*8*	≥*16*				*0.5*
	*S. chromogenes*	6						100.0									
	*S. epidermidis*	27						100.0									
	*S. haemolyticus*	5						100.0									
	*S. simulans*	30	1 (3.3)					96.7	3.3								
Clindamycin							≤*0.25*	*0.5*	*1*	*2*	*4*	≥*8*					*0.25*
	*S. chromogenes*	6					100.0										
	*S. epidermidis*	27					100.0										
	*S. haemolyticus*	5					100.0										
	*S. simulans*	30	3 (10.0)				90.0	10.0									
Erythromycin							≤*0.25*	*0.5*	*1*	*2*	*4*	≥*8*					*1.0*
	*S. chromogenes*	6						83.3	16.7								
	*S. epidermidis*	27					25.9	70.4	3.7								
	*S. haemolyticus*	5					20.0	80.0									
	*S. simulans*	30					10.0	86.7	3.3								
Chloramphenicol										≤*2*	*4*	*8*	*16*	≥*32*			*16.0*
	*S. chromogenes*	6									33.3	66.7					
	*S. epidermidis*	27									92.6	7.4					
	*S. haemolyticus*	5									100.0						
	*S. simulans*	30									76.7	23.3					
Tetracycline								≤*0.5*	*1*	*2*	*4*	*8*	*16*	*32*	*64*	≥*128*	*1.0*
	*S. chromogenes*	6						100.0									
	*S. epidermidis*	27	7 (25.9)					66.7	7.4	18.5					7.4		
	*S. haemolyticus*	5						100.0									
	*S. simulans*	30	2 (6.7)					90.0	3.3				6.7				
Trimetoprim-sulfamethoxazole								≤*0.5/9.5*	*1/19*	*2/38*	≥*4/76*						*0.5/9.5*
	*S. chromogenes*	6						100.0									
	*S. epidermidis*	27	2 (7.4)					92.6			7.4						
	*S. haemolyticus*	5						100.0									
	*S. simulans*	30						100.0									
Cefoxitin						≤*0.12*	*0.25*	*0.5*	*1*	*2*	*4*	*8*	*16*	≥*32*			*4.0* ^b^
	*S. chromogenes*	6						83.3	16.7								
	*S. epidermidis*	27	5 (18.5)						3.7	55.6	22.2	7.4	3.7	7.4			
	*S. haemolyticus*	5								40.0	60.0						
	*S. simulans*	30						3.3		80.0	16.7						
Kanamycin						≤*0.12*	*0.25*	*0.5*	*1*	*2*	*4*	*8*	*16*	*32*	≥*64*		*8.0* ^b^
	*S. chromogenes*	6						33.3	66.7								
	*S. epidermidis*	27	2 (7.4)					18.5	48.1	25.9			3.7		3.7		
	*S. haemolyticus*	5						80.0	20.0								
	*S. simulans*	30						30.0	40.0	30.0							
Florphenicol										≤*2*	*4*	*8*	≥*16*				*8.0* ^b^
	*S. chromogenes*	6								50.0	50.0						
	*S. epidermidis*	27								48.1	51.9						
	*S. haemolyticus*	5								60.0	40.0						
	*S. simulans*	30								10.0	86.7	3.3					
Trimetoprim								≤*0.5*	*1*	*2*	*4*	*8*	*16*	*32*	≥*64*		*2.0* ^b^
	*S. chromogenes*	6							50.0	50							
	*S. epidermidis*	27	4 (14.8)					11.1	44.4	29,6		3.7	3.7		7.4		
	*S. haemolyticus*	5	4 (80.0)							20.0	40.0		40.0				
	*S. simulans*	30	29 (96.7)							3.3	6.7	36.7	50.0	3.3			
Ciprofloxacin					≤*0.06*	*0.12*	*0.25*	*0.5*	≥*1*								*1.0*
	*S. chromogenes*	6				100.0											
	*S. epidermidis*	27				14.8	81.5	3.7									
	*S. haemolyticus*	5					80.0	20.0									
	*S. simulans*	30				50.0	50.0										
Fusidic acid					≤*0.06*	*0.12*	*0.25*	*0.5*	*1*	*2*	*4*	*8*	≥*16*				*0.5*
	*S. chromogenes*	6	1 (16.7)				66.7	16.7	16.7								
	*S. epidermidis*	27	10 (37.0)				3.7	59.3	25.9			3.7	7.4				
	*S. haemolyticus*	5						100.0									
	*S. simulans*	30	10 (33.3)				6.7	60.0	33.3								

In this dataset, samples were collected in the Finnish veterinary antimicrobial resistance monitoring program 2010-2012 [18] and they originated from quarters with mastitis

^a^Current (March 2015) EUCAST epidemiological cut-off (ECOFF) values (mg/l) for CNS were used to define resistant isolates. If ECOFF for CNS was not available, the value for *S. aureus* was used. For virginiamycin, ECOFF for *S. intermedius* was used

^b^ECOFF for *S. aureus*

The majority, 74.4 and 74.1 % of *S. epidermidis* isolates in years 2001 and 2012, respectively, were resistant to benzylpenicillin (ECOFF 0.125 mg/l). Resistance to benzylpenicillin was also common in *S. haemolyticus* of which 64.9/40.0 % were resistant. Prevalence of isolates producing betalactamase, i.e. positive in the nitrocefin test, varied between CoNS species, and was lower in *S. chromogenes* (20.8/16.7 %) and *S. simulans* (3.9/6.7 %) than in *S. epidermidis* (59.0/70.4 %) and *S. haemolyticus* (51.4/0.0 %). Combining years 2001 and 2012, a total of 165 of the total of 400 isolates (41.3 %) had a MIC > 0.125 mg/l for benzylpenicillin i.e. were penicillin-resistant. In the nitrocefin test, 137 (34.3 %) isolates out of 400 were positive (penicillin resistant). Out of 165 isolates with MIC for > 0.125 mg/l for benzylpenicillin, 127 (77.0 %) isolates were positive in the nitrocefin test (true positive), and 38 were negative (false negative). Out of 235 isolates with MIC ≤ 0.125 mg/l for benzylpenicillin, 225 isolates (95.7 %) were negative in the nitrocefin test (true negative), and 10 isolates were positive (false positive). The positive predictive value of the nitrocefin test in relation to penicillin resistance determined based on the MIC value of the isolate was 92.7 and the negative predictive value 85.6.

Considerable proportions of *S. epidermidis* were resistant to tetracycline (46.2/25.9 %) and streptomycin (21.8/7.4 %) (years 2001/2012). In the other species this resistance was rare (Tables 1, 2). Oxacillin resistance (conferring resistance also to methicillin), using ECOFF of 1.0 mg/l, was found in all the four main species (Tables 2, 3). Combining years 2001 and 2012, a total of 137 isolates (34.3 %) were oxacillin resistant. The *mec*A gene was detected in 16 *S. epidermidis* isolates (20.5 %) from the year 2001 and in four *S. epidermidis* isolates (14.8 %) from the year 2012, and in the one *S. sciuri* isolate. Isolates harboring *mec*C were not found. Resistance to trimethoprim, measured only in dataset 2, was most common in *S. simulans* and *S. haemolyticus*, but the number of isolates of the latter species was low (Table 3). *Staphylococcus epidermidis* was the only species showing resistance to cefoxitin using the ECOFF of *S. aureus* (4 mg/l) (Table 3). Resistance to fusidic acid was common in all other species but *S. haemolyticus* (Table 3). One *S. simulans* isolate was resistant to vancomycin, using the specific ECOFF (4 mg/l) for that species (Table 2).

Combining datasets 1 and 2, a total of 44.4 % of *S. chromogenes*, 23.8 % of *S. haemolyticus*, 45.4 % of *S. simulans* and 16.2 % of *S. epidermidis* isolates were susceptible to all antimicrobials tested. Resistance to more than one antimicrobial was most common in *S. epidermidis* isolates. Close to one-third (28.6 %) of them were resistant to >2 antimicrobials. Among the other three major species, one *S. chromogenes* isolate, one *S. haemolyticus* isolate, and five *S. simulans* isolates (4.6 %) were resistant to >2 substances. Seven *S. epidermidis* isolates (6.7 %) were multidrug-resistant (MDR = resistant to 3 or more classes of antimicrobials); one isolate was resistant to five different antimicrobial classes. Among the other species, only one MDR *S. simulans* isolate was found.

## Discussion

The most common CoNS species in our data, *S. simulans*, *S. epidermidis*, *S. chromogenes*, and *S. haemolyticus,* belong to the CoNS species reported most frequently in numerous studies on bovine intramammary infection (IMI) or mastitis. *Staphylococcus chromogenes* has been isolated most commonly in almost all studies [11–13, 25–29]. It is much more common in primiparous than multiparous cows [11, 28, 30], with peak occurrence around the first calving [4]. *Staphylococcus chromogenes* seems to be present in all herds [26–29] and has been frequently isolated not only from milk but also from bovine teat skin and orifice and from other body sites of heifers and cows [20, 31]. *Staphylococcus simulans* and *S. epidermidis* are common causes of IMI in some herds but not found or only occasionally found in some other herds [26, 27, 29, 30, 32]. Both *S. epidermidis* and *S. simulans* are reported to be more common in IMIs of multiparous than primiparous cows [11, 28, 30]. For some reason, *S. simulans* is common in the Nordic countries [11, 12, 33] but not so much in Middle European countries [12, 25, 26]. *Staphylococcus xylosus* is commonly reported in Dutch and Belgian studies [13, 26, 27] but is rare in Finland, Norway and Sweden [6, 11, 28]. *Staphylococcus haemolyticus* is a fairly common finding in many studies [11, 13, 26–29]. The reasons for variable proportions of CoNS species isolated from dairy cattle in different countries and individual herds are not fully elucidated but are likely related to different environmental conditions and herd management [10, 31].

In the present study, genotypic identification of CoNS species and species-specific or CoNS-specific EUCAST ECOFFs when available were used. In only few other studies species-specific identification and the same cut-offs than here have been used [5, 6]. In a Swedish study, prevalence of resistant isolates in the four major CoNS species was substantially lower than here [6]. Our results agree with them in that the most common resistance was to benzylpenicillin, but proportion of resistant isolates was clearly lower in the Swedish study. Only one out of 34 Swedish *S. epidermidis* isolates was resistant to oxacillin and harboured the *mec*A gene. In a Swiss study [5], oxacillin resistance was the most common resistance phenotype. They found as much as 47.0 % of the isolates (all CoNS together) to be oxacillin resistant, but the cut-off used was two dilutions lower (0.25 mg/l) than the current EUCAST ECOFF, which explains the discrepant results. The *mec*A gene was present in 9.7 % of the isolates classified as oxacillin-resistant. In the study by Frey et al. [5] the total proportion of penicillin-resistant isolates was lower (23.3 %) than in our study, using the same ECOFF, but the selection of CoNS species was different from ours. Results from the nitrocefin test were compared with results based on the penicillin MIC values of the isolates. The nitrocefin test performed better in detecting penicillin susceptible CoNS isolates; of isolates with MIC for benzylpenicillin >0.125 mg/l, considered as resistant, 23 % were negative in the test. These results agree with the study by Pitkälä et al. [34] comparing different betalactamase tests, who found no false positive but some false negative results for *bla*Z positive CoNS using nitrocefin disk test. In testing bovine *S. aureus* isolates, nitrocefin test has been found to be very reliable [34, 35]. Frey et al. [5] carried out in vitro betalactamase testing with pre-incubation with benzylpenicillin but did not report the results, so we cannot compare them with ours. Interpreting results of bovine CoNS isolates from nitrocefin disk assay is challenging, as the color change is sometimes slow and not clear (Suvi Nykäsenoja, Evira, personal communication). The performance of the nitrocefin tests may not be fully satisfactory in detecting penicillin resistance of bovine CoNS. This is of practical importance because nitrocefin tests are widely used to predict betalactamase production of bovine staphylococci.

*Staphylococcus epidermidis* was the most resistant among the four major species identified. Almost one-third of our *S. epidermidis* isolates were resistant to >2 antimicrobials and close to 7 % were MDR, which were not found among the other species. This agrees with previous studies which also have reported *S. epidermidis* being frequently resistant to several antimicrobials [5, 36]. The most common combination was resistance to penicillin and tetracycline. All *mec*A positive isolates, except one *mec*A positive *S. sciuri* isolate, were *S. epidermidis*. However, phenotypic methicillin (oxacillin) resistance was also common in the other CoNS species. Among the other three major species, MIC values to oxacillin in the methicillin-resistant isolates were mainly only one step higher than ECOFF and did not form a distinctly different population with clearly higher MIC values. According to many studies, methicillin-resistance is much more common in *S. epidermidis* than in other CoNS species [8, 36–38]. Among clinical *S. epidermidis* isolates from humans, 75–90 % are resistant to methicillin (reviewed by Otto [39]). *Staphylococcus epidermidis* differs from other mastitis causing CoNS in many aspects. It is a well-known human pathogen which causes nosocomial infections often associated with medical devices [39, 40]. *Staphylococcus epidermidis* has a selection of virulence characteristics which include biofilm formation and antimicrobial resistance [3, 40]. It has been suggested that bovine *S. epidermidis* may originate from humans [38, 41]. A Finnish study did not find bovine methicillin-resistant *S. epidermidis* strains being closely related to human isolates [8].

Nearly all *S. simulans* isolates were resistant to trimethoprim. No specific ECOFF is available for this antimicrobial, and we used that of *S. aureus*. The MICs of trimethoprim of most *S. simulans* isolates were several steps above the ECOFF used, which indicates true resistance. More than one-third of *S. epidermidis* and *S. simulans* were resistant to fusidic acid according to ECOFF for CoNS. Looking at the MIC distributions of our isolates, this ECOFF seemed not optimal for this group of CoNS. In a Norwegian study, 10 % of bovine CoNS isolates were resistant to fusidic acid using the same ECOFF, but all isolates classified as resistant had MIC values several steps over the cut-off [42]. In some CoNS species resistance is more common (Table 2). Almost half of *S. epidermidis* isolates of human origin have been resistant to fusidic acid and harbored the same horizontally acquired resistance determinants than reported in *S. aureus* [43].

Antimicrobial resistance among bovine CoNS isolates causes two types of concerns. First, it decreases options for antimicrobial treatment of mastitis as well as response to treatment. Mastitis caused by CoNS is mostly subclinical or mild clinical and routine treatment is not recommended [4]. In cases where treatment is warranted, resistance to benzylpenicillin is an issue at least in countries where penicillin or aminopenicillins are the drugs of choice [4]. Penicillin resistance of three of the four major CoNS species was here at so high level that penicillin can no more be considered as the first treatment option in mastitis caused by these species. Another option could be macrolides to which some degree of resistance was also found, in particular among *S. epidermidis* isolates. Prevalences of oxacillin resistant isolates were alarmingly high in all four major species. Cloxacillin is commonly used to treat mastitis, also mastitis caused by CoNS, so this is of practical relevance. If the causing CoNS strain harbors a *mec* gene, treatment with any betalactam antimicrobial is inefficient and only increases selection pressure.

The second concern is related to public health: cows can pass resistant CoNS to humans via direct contact or indirectly [37]. Bovine CoNS can also act as a reservoir for resistance determinants [3, 44]. The greatest concern is methicillin-resistance which was common in bovine CoNS and presents a relevant risk for public health [38, 40]. There is evidence for transfer of resistance determinants between staphylococcal species and also from CoNS to the more pathogenic species *S. aureus* [9, 44–46]. Regarding critically important antimicrobials [47] included in the present study, the situation was good as only one CoNS isolate (*S. simulans*) was resistant to vancomycin and no isolates to ciprofloxacin.

Unfortunately specific ECOFFs are not yet available for all CoNS species, and we had to use those of *S. aureus* for several antimicrobials. EUCAST ECOFFs are not veterinary specific but isolates originate from multiple sources and perhaps mainly from humans. Most studies on antimicrobial resistance of CoNS have used CLSI (Clinical and Laboratory Standards Institute) [48] breakpoints for veterinary pathogens. CLSI breakpoints are aimed for clinical purposes only [48]. They are derived from animal specific microbiological, pharmacokinetic and pharmacodynamic data, which is not relevant for studies on in vitro susceptibility of epidemiological data sets [15]. Furthermore, animal specific breakpoints are not available for all antimicrobials and those based on human data have been used [48]. CLSI documents do not give specific breakpoints for bovine CoNS, but some breakpoints are available for *S. aureus*. In general, CLSI breakpoints are higher than EUCAST ECOFFs. Studies which have used different breakpoints for resistance cannot be compared. After phenotypic screening of antimicrobial resistance, as done here, the next step would be to study the genetic mechanisms for resistance. For many antimicrobials several genes can code for resistance in staphylococci, and new genes are discovered [44, 45]. Selection of a representative panel of resistance genes for genotypic studies is challenging.

## Conclusions

The most common CoNS species were *S. simulans*, *S. epidermidis*, *S. chromogenes*, and *S. haemolyticus.* *Staphylococcus epidermidis* differed from the three other most common CoNS species isolated from mastitic bovine milk samples as resistance to most tested antimicrobials was more common in *S. epidermidis* than in *S. chromogenes*, *S. haemolyticus* or *S. simulans*. Except one *S. sciuri* isolate, all *mec*A gene positive isolates were *S. epidermidis.* Resistance to more than two antimicrobials was also common in *S. epidermidis*.

## Additional files

**Additional file 1: Table S1.** In vitro susceptibility to 20 antimicrobials of coagulase-negative *Staphylococcus* isolates from bovine milk samples from dataset 1 (2001, 312 isolates) [2] and 2 (2012, 88 isolates) [18] (pooled data).

**Additional file 2: Table S2.** In vitro susceptibility to 20 antimicrobials of coagulase-negative *Staphylococcus* isolates from bovine milk samples from dataset 1 (2001, 312 isolates) [2] and 2 (2012, 88 isolates) [18].

## References

[1] Myllys V, Asplund K, Brofeldt E, Hirvelä-Koski V, Honkanen-Buzalski T, Junttila J (1998). Bovine mastitis in Finland in 1988 and 1995—changes in prevalence and antimicrobial resistance. Acta Vet Scand.

[2] Pitkälä A, Haveri M, Pyörälä S, Myllys V, Honkanen-Buzalski T (2004). Bovine mastitis in Finland 2001—prevalence, distribution of bacteria, and antimicrobial resistance. J Dairy Sci.

[3] Vanderhaeghen W, Piepers S, Leroy F, Van Coillie E, Haesebrouck F, De Vliegher S (2014). Invited review: effect, persistence, and virulence of coagulase-negative *Staphylococcus* species associated with ruminant udder health. J Dairy Sci.

[4] Pyörälä S, Taponen S (2009). Coagulase-negative staphylococci—emerging mastitis pathogens. Vet Microbiol.

[5] Frey Y, Rodriguez JP, Thomann A, Schwendener S, Perreten V (2013). Genetic characterization of antimicrobial resistance in coagulase-negative staphylococci from bovine mastitis milk. J Dairy Sci.

[6] Persson Waller K, Aspán A, Nyman A, Persson Y, Grönlund Andersson U (2011). CNS species and antimicrobial resistance in clinical and subclinical bovine mastitis. Vet Microbiol.

[7] Huber H, Ziegler D, Pflüger V, Vogel G, Zweifel C, Stephan R (2011). Prevalence and characteristics of methicillin resistant coagulase-negative staphylococci from livestock, chicken carcasses, bulk tank milk, minced meat, and contact persons. BMC Vet Res.

[8] Gindonis V, Taponen S, Myllyniemi A-M, Pyörälä S, Nykäsenoja S, Salmenlinna S (2013). Occurrence and characterization of methicillin-resistant staphylococci from bovine mastitis milk samples in Finland. Acta Vet Scand.

[9] Juuti K, Ibrahem S, Virolainen-Julkunen A, Vuopio-Varkila J, Kuusela P (2005). The pls gene found in methicillin-resistant *Staphylococcus aureus* strains is common in clinical isolates of *Staphylococcus sciuri*. J Clin Microbiol.

[10] Vanderhaeghen W, Vandendriessche S, Crombé F, Nemeghaire S, Dispas M, Denis O (2013). Characterization of methicillin-resistant non-*Staphylococcus aureus* staphylococci carriage isolates from different bovine populations. J Antimicrob Chemother.

[11] Taponen S, Simojoki H, Haveri M, Larsen HD, Pyörälä S (2006). Clinical characteristics and persistence of bovine mastitis caused by different species of coagulase-negative staphylococci identified with API or AFLP. Vet Microbiol.

[12] Capurro A, Artursson K, Persson Waller K, Bengtsson B, Ericsson-Unnerstad H, Aspán A (2009). Comparison of a commercialized phenotyping system, antimicrobial susceptibility testing, and a *tuf* gene sequence-based genotyping for species-level identification of coagulase-negative staphylococci isolated from cases of bovine mastitis. Vet Microbiol.

[13] Sampimon OC, Zadoks RN, De Vliegher S, Supré K, Haesebrouck F, Barkema HW (2009). Performance of API Staph ID 32 and Staph-Zym for identification of coagulase-negative staphylococci isolated from bovine milk samples. Vet Microbiol.

[14] Moser A, Stephan R, Ziegler D, Johler S (2013). Species distribution and resistance profiles of coagulase-negative staphylococci isolated from bovine mastitis in Switzerland. Schweiz Arch Tierheilkd.

[15] Schwarz S, Silleya P, Simjee S, Woodford N, van Duijkeren E, Johnson AP (2010). Editorial: assessing the antimicrobial susceptibility of bacteria obtained from animals. Vet Microbiol.

[16] Ruegg PL, Oliveira L, Jin W, Okwumabua O (2015). Phenotypic antimicrobial susceptibility and occurrence of selected resistance genes in gram-positive mastitis pathogens isolated from Wisconsin dairy cows. J Dairy Sci.

[17] Wendlandt S, Kadlec K, Feßler AT, Schwarz S (2015). Identification of ABC transporter genes conferring combined pleuromutilin–lincosamide–streptogramin A resistance in bovine methicillin-resistant *Staphylococcus aureus* and coagulase-negative staphylococci. Vet Microbiol.

[18] FINRES-Vet 2010–2012. Finnish Veterinary Antimicrobial Resistance Monitoring and Consumption of Antimicrobial Agents. Evira publications 2/2015. Finnish Food Safety Authority Evira, Helsinki, Finland. ISSN 1797-299X, ISBN 978-952-225-143-5 (pdf). http://www.evira.fi/portal/fi/tietoa+evirasta/julkaisut/?a=view&productId=412 .

[19] Honkanen-Buzalski T, Seuna E. Isolation and identification of pathogens from milk. In: Sandholm M, Honkanen-Buzalski T, Kaartinen L, Pyörälä S, editors. The bovine udder and mastitis. Gummerus, Jyväskylä, Finland; 1995. p. 121–41.

[20] Taponen S, Björkroth J, Pyörälä S (2008). Coagulase-negative staphylococci isolated from bovine extramammary sites and intramammary infections in a single dairy herd. J Dairy Res.

[21] Dubois D, Leyssene D, Chacornac JP, Kostrzewa M, Schmit PO, Talon R (2010). Identification of a variety of *Staphylococcus* species by matrix-assisted laser desorption ionization-time of flight mass spectrometry. J Clin Microbiol.

[22] EUCAST: European Committee on Antimicrobial Susceptibility testing. European Society of Clinical Microbiology and Infectious Diseases. http://www.eucast.org/. 10.1111/j.1469-0691.2006.01454.x 16700696

[23] Murakami K, Minamide W, Wada K, Nakamura E, Teraoka H, Watanabe S (1991). Identification of methicillin-resistant strains of staphylococci by polynerase chain reaction. J Clin Microbiol.

[24] Stegger M, Andersen PS, Kearns A, Pichon B, Holmes MA, Edwards G (2012). Rapid detection, differentiation and typing of methicillin-resistant *Staphylococcus aureus* harbouring either *mecA* or the new *mecA* homologue *mecA* (LGA251). Clin Microbiol Infect.

[25] Rajala-Schultz PJ, Torres AH, DeGraves FJ, Gebreyes WA, Patchanee P (2009). Antimicrobial resistance and genotypic characterization of coagulase-negative staphylococci over the dry period. Vet Microbiol.

[26] Piessens V, Van Coillie E, Verbist B, Supré K, Braem G, Van Nuffel A (2011). Distribution of coagulase-negative *Staphylococcus* species from milk and environment of dairy cows differs between herds. J Dairy Sci.

[27] Supré K, Haesebrouck F, Zadoks RN, Vaneechoutte M, Piepers S, De Vliegher S (2011). Some coagulase-negative *Staphylococcus* species affect udder health more than others. J Dairy Sci.

[28] Mørk T, Jørgensen HJ, Sunde M, Kvitle B, Sviland S, Waage S (2012). Persistence of staphylococcal species and genotypes in the bovine udder. Vet Microbiol.

[29] Bexiga R, Rato MG, Lemsaddek A, Semedo-Lemsaddek T, Carneiro C, Pereira H (2014). Dynamics of bovine intramammary infections due to coagulase-negative staphylococci on four farms. J Dairy Res.

[30] Thorberg BM, Danielsson-Tham ML, Emanuelson U (2009). Persson Waller K. Bovine subclinical mastitis caused by different types of coagulase-negative staphylococci. J Dairy Sci.

[31] De Visscher A, Piepers S, Haesebrouck F, De Vliegher S (2016). Teat apex colonization with coagulase-negative *Staphylococcus* species before parturition: distribution and species-specific risk factors. J Dairy Sci.

[32] Gillespie BE, Headrick SI, Boonyayatra S, Oliver SP (2009). Prevalence and persistence of coagulase-negative *Staphylococcus* species in three dairy research herds. Vet Microbiol.

[33] Waage S, Mørk T, Røros A, Aasland D, Hunshamar A, Ødegaard SA (1999). Bacteria associated with clinical mastitis in dairy heifers. J Dairy Sci.

[34] Pitkälä A, Salmikivi L, Bredbacka P, Myllyniemi A-L, Koskinen MT (2007). Comparison of tests for detection of ß-lactamase-producing Staphylococci. J Clin Microbiol.

[35] Haveri M, Suominen S, Rantala L, Honkanen-Buzalski T, Pyörälä S (2005). Comparison of phenotypic and genotypic detection of penicillin G resistance of *Staphylococcus aureus* isolated from bovine intramammary infection. Vet Microbiol.

[36] Sampimon OC, Lam TJ, Mevius DJ, Schukken YH, Zadoks RN (2011). Antimicrobial susceptibility of coagulase-negative staphylococci isolated from bovine milk samples. Vet Microbiol.

[37] Feßler AT, Billerbeck C, Kadlec K, Schwarz S (2010). Identification and characterization of methicillin-resistant coagulase-negative staphylococci from bovine mastitis. J Antimicrob Chemother.

[38] Jaglic Z, Michu E, Holasova M, Vlkova H, Babak V, Kolar M (2010). Epidemiology and characterization of *Staphylococcus epidermidis* isolates from humans, raw bovine milk and a dairy plant. Epidemiol Infect.

[39] Otto M (2009). *Staphylococcus epidermidis*—the “accidental” pathogen. Nat Rev Microbiol.

[40] Schoenfelder SMK, Langea C, Eckart M, Hennig S, Kozytska S, Ziebuhr W (2010). Success through diversity—how *Staphylococcus epidermidis* establishes as a nosocomial pathogen. Int J Med Microbiol.

[41] Thorberg B-M, Kühn I, Aarestrup FM, Brändström B, Jonsson P, Danielsson-Tham M-L (2006). Pheno- and genotyping of *Staphylococcus epidermidis* isolated from bovine milk and human skin. Vet Microbiol.

[42] Yazdankdhah SP, Åsli AW, Sørum H, Oppegaard H, Sunde M (2006). Fusidic acid resistance, mediated by *fus*B, in bovine coagulase-negative staphylococci. J Antimicrob Chemother.

[43] McLaws F, Chopra I, O’Neill AJ (2008). High prevalence of resistance to fusidic acid in clinical isolates of *Staphylococcus epidermidis*. J Antimicrob Chemother.

[44] Nemeghaire S, Argudín MA, Feßler AT, Hauschild T, Schwarz S, Butaye P (2014). The ecological importance of the *Staphylococcus sciuri* species group as a reservoir for resistance and virulence genes. Vet Microbiol.

[45] Wendlandt S, Feßler AT, Monecke S, Ehricht R, Schwarz S, Kadlec K (2013). The diversity of antimicrobial resistance genes among staphylococci of animal origin. Int J Med Microbiol.

[46] Zhang Y, Agidi S, LeJeune JT (2009). Diversity of staphylococcal cassette chromosome in coagulase-negative staphylococci from animal sources. J Appl Microbiol.

[47] Collignon P, Powers J, Chiller T, Aidara-Kane A, Aarestrup F (2009). World Health Organization ranking of antimicrobials according to their importance in human medicine: a critical step for developing risk management strategies for the use of antimicrobials in food production animals. Clin Inf Dis..

[48] CLSI. VET01-S2. Performance standards for Antimicrobial disk and dilution susceptibility test for bacteria isolated from animals; Second Informational Supplement. Clinical and Laboratory Standards Institute, 950 West Valley Road, Suite 2500, Wayne; 2013.

