# Supplementary material for: Species distribution and in vitro antimicrobial susceptibility of coagulase-negative staphylococci isolated from bovine mastitic milk
Source: Acta Vet Scand. 2016 Feb 6;58:12. doi: 10.1186/s13028-016-0193-8 (PMC4744398; doi:10.1186/s13028-016-0193-8)
Supplement: Supplementary file 1 — Additional file 1: Table S1. In vitro susceptibility to 20 antimicrobials of coagulase-negative Staphylococcus isolates from bovine milk samples from dataset 1 (2001, 312 isolates) [2] and 2 (2012, 88 isolates) [18] (pooled data). [file 13028_2016_193_MOESM1_ESM.docx]

Table S1. *In vitro* susceptibility to 20 antimicrobials of coagulase-negative *Staphylococcus* isolates from bovine milk samples from dataset 1 (2001, 312 isolates) [2] and 2 (2012, 88 isolates) [18] (pooled data).

|  | **Organism** | **N** | **% >ECOFF** | Number of isolates with indicated MIC, mg/l | | | | | | | | | | | | | | | **ECOFF*** |
| --- | --- | --- | --- | --- | --- | --- | --- | --- | --- | --- | --- | --- | --- | --- | --- | --- | --- | --- | --- |
| Benzylpenicillin |  |  |  |  |  | **≤0.06** | **0.12** | **0.25** | **0.5** | **1** | **2** | **4** | **≥8** |  |  |  |  |  | **0.125^†^** |
|  | *S. agnetis* | 10 |  |  |  | 9 | 1 |  |  |  |  |  |  |  |  |  |  |  |  |
|  | *S. capitis* | 2 | 50.0 |  |  |  | 1 | 1 |  |  |  |  |  |  |  |  |  |  |  |
|  | *S. chromogenes* | 54 | 27.8 |  |  | 38 | 1 | 2 | 3 | 7 | 3 |  |  |  |  |  |  |  |  |
|  | *S. cohnii* | 12 | 75.0 |  |  |  | 3 | 8 | 1 |  |  |  |  |  |  |  |  |  |  |
|  | *S. epidermidis* | 105 | 74.3 |  |  | 24 | 3 | 8 | 9 | 15 | 14 | 13 | 19 |  |  |  |  |  |  |
|  | *S. equorum* | 3 |  |  |  |  | 3 |  |  |  |  |  |  |  |  |  |  |  |  |
|  | *S. haemolyticus* | 42 | 61.9 |  |  | 15 | 1 | 6 | 10 | 7 | 2 | 1 |  |  |  |  |  |  |  |
|  | *S. hyicus* | 5 |  |  |  | 4 | 1 |  |  |  |  |  |  |  |  |  |  |  |  |
|  | *S. kloosii* | 3 | 66.7 |  |  | 1 |  | 2 |  |  |  |  |  |  |  |  |  |  |  |
|  | *S. pasteuri* | 2 | 50.0 |  |  | 1 |  |  |  |  | 1 |  |  |  |  |  |  |  |  |
|  | *S. saprophyticus* | 3 | 66.7 |  |  | 1 |  | 1 | 1 |  |  |  |  |  |  |  |  |  |  |
|  | *S. sciuri* | 3 | 100.0 |  |  |  |  | 1 | 2 |  |  |  |  |  |  |  |  |  |  |
|  | *S. simulans* | 108 | 4.6 |  |  | 101 | 2 | 2 |  | 1 | 2 |  |  |  |  |  |  |  |  |
|  | *S. warneri* | 33 | 45.5 |  |  | 13 | 2 | 2 | 5 | 4 | 5 | 1 | 1 |  |  |  |  |  |  |
|  | *S. xylosus* | 10 | 50.0 |  |  |  | 5 | 4 |  | 1 |  |  |  |  |  |  |  |  |  |
|  | *S.* sp | 5 | 40.0 |  |  | 2 | 1 | 1 | 1 |  |  |  |  |  |  |  |  |  |  |
| Oxacillin |  |  |  |  |  |  |  |  | **≤0.5** | **1** | **2** | **4** | **≥8** |  |  |  |  |  | **1.0** |
|  | *S. agnetis* | 10 | 60.0 |  |  |  |  |  | 3 | 1 | 6 |  |  |  |  |  |  |  |  |
|  | *S. capitis* | 2 | 50.0 |  |  |  |  |  |  | 1 | 1 |  |  |  |  |  |  |  |  |
|  | *S. chromogenes* | 54 | 35.2 |  |  |  |  |  | 16 | 19 | 18 | 1 |  |  |  |  |  |  |  |
|  | *S. cohnii* | 12 | 100.0 |  |  |  |  |  |  |  | 3 | 8 | 1 |  |  |  |  |  |  |
|  | *S. epidermidis* | 105 | 29.5 |  |  |  |  |  | 49 | 25 | 12 | 6 | 13 |  |  |  |  |  |  |
|  | *S. equorum* | 3 | 66.7 |  |  |  |  |  |  | 1 | 2 |  |  |  |  |  |  |  |  |
|  | *S. haemolyticus* | 42 | 23.8 |  |  |  |  |  | 16 | 16 | 9 | 1 |  |  |  |  |  |  |  |
|  | *S. hyicus* | 5 | 80.0 |  |  |  |  |  |  | 4 | 1 |  |  |  |  |  |  |  |  |
|  | *S. kloosii* | 3 | 100.0 |  |  |  |  |  |  |  | 3 |  |  |  |  |  |  |  |  |
|  | *S. pasteuri* | 2 | 50.0 |  |  |  |  |  | 1 |  | 1 |  |  |  |  |  |  |  |  |
|  | *S. saprophyticus* | 3 | 100.0 |  |  |  |  |  |  |  | 2 | 1 |  |  |  |  |  |  |  |
|  | *S. sciuri* | 3 | 100.0 |  |  |  |  |  |  |  |  | 2 | 1 |  |  |  |  |  |  |
|  | *S. simulans* | 108 | 15.7 |  |  |  |  |  | 43 | 48 | 16 | 1 |  |  |  |  |  |  |  |
|  | *S. warneri* | 33 | 48.5 |  |  |  |  |  | 5 | 12 | 13 | 3 |  |  |  |  |  |  |  |
|  | *S. xylosus* | 10 | 80.0 |  |  |  |  |  |  | 2 | 5 | 2 | 1 |  |  |  |  |  |  |
|  | *S.* sp | 5 | 75.0 |  |  |  |  |  | 1 |  | 3 | 1 |  |  |  |  |  |  |  |
| Cephalothin |  |  |  |  |  |  | **≤0.12** | **0.25** | **0.5** | **1** | **2** | **4** | **≥8** |  |  |  |  |  | **1.0^†^** |
|  | *S. agnetis* | 10 |  |  |  |  | 1 | 9 |  |  |  |  |  |  |  |  |  |  |  |
|  | *S. capitis* | 2 |  |  |  |  |  | 2 |  |  |  |  |  |  |  |  |  |  |  |
|  | *S. chromogenes* | 54 |  |  |  |  | 14 | 37 | 3 |  |  |  |  |  |  |  |  |  |  |
|  | *S. cohnii* | 12 |  |  |  |  |  |  | 2 |  |  |  |  |  |  |  |  |  |  |
|  | *S. epidermidis* | 105 |  |  |  |  | 29 | 53 | 10 | 13 |  |  |  |  |  |  |  |  |  |
|  | *S. equorum* | 3 |  |  |  |  |  |  | 2 | 1 |  |  |  |  |  |  |  |  |  |
|  | *S. haemolyticus* | 42 |  |  |  |  | 5 | 27 | 10 |  |  |  |  |  |  |  |  |  |  |
|  | *S. hyicus* | 5 |  |  |  |  |  | 4 | 1 |  |  |  |  |  |  |  |  |  |  |
|  | *S. kloosii* | 3 |  |  |  |  |  | 1 | 2 |  |  |  |  |  |  |  |  |  |  |
|  | *S. pasteuri* | 2 |  |  |  |  |  | 2 |  |  |  |  |  |  |  |  |  |  |  |
|  | *S. saprophyticus* | 3 |  |  |  |  |  | 1 | 2 |  |  |  |  |  |  |  |  |  |  |
|  | *S. sciuri* | 3 |  |  |  |  |  | 1 | 2 |  |  |  |  |  |  |  |  |  |  |
|  | *S. simulans* | 108 |  |  |  |  | 7 | 65 | 36 |  |  |  |  |  |  |  |  |  |  |
|  | *S. warneri* | 33 | 3.0 |  |  |  | 4 | 20 | 6 | 2 | 1 |  |  |  |  |  |  |  |  |
|  | *S. xylosus* | 10 | 20.0 |  |  |  |  | 4 | 3 | 1 | 2 |  |  |  |  |  |  |  |  |
|  | *S.* sp | 5 |  |  |  |  | 1 | 1 | 2 | 1 |  |  |  |  |  |  |  |  |  |
| Streptomycin |  |  |  |  |  |  |  |  |  |  |  | **≤4** | **8** | **16** | **32** | **≥64** |  |  | **16.0^†^** |
|  | *S. agnetis* | 10 |  |  |  |  |  |  |  |  |  | 8 | 2 |  |  |  |  |  |  |
|  | *S. capitis* | 2 |  |  |  |  |  |  |  |  |  | 1 | 1 |  |  |  |  |  |  |
|  | *S. chromogenes* | 54 |  |  |  |  |  |  |  |  |  | 51 | 2 | 1 |  |  |  |  |  |
|  | *S. cohnii* | 12 |  |  |  |  |  |  |  |  |  | 12 |  |  |  |  |  |  |  |
|  | *S. epidermidis* | 105 | 18.1 |  |  |  |  |  |  |  |  | 80 | 5 | 1 | 3 | 16 |  |  |  |
|  | *S. equorum* | 3 |  |  |  |  |  |  |  |  |  | 3 |  |  |  |  |  |  |  |
|  | *S. haemolyticus* | 37 |  |  |  |  |  |  |  |  |  | 42 |  |  |  |  |  |  |  |
|  | *S. hyicus* | 5 |  |  |  |  |  |  |  |  |  | 4 | 1 |  |  |  |  |  |  |
|  | *S. kloosii* | 3 |  |  |  |  |  |  |  |  |  | 3 |  |  |  |  |  |  |  |
|  | *S. pasteuri* | 2 |  |  |  |  |  |  |  |  |  | 2 |  |  |  |  |  |  |  |
|  | *S. saprophyticus* | 3 |  |  |  |  |  |  |  |  |  | 3 |  |  |  |  |  |  |  |
|  | *S. sciuri* | 3 |  |  |  |  |  |  |  |  |  | 2 | 1 |  |  |  |  |  |  |
|  | *S. simulans* | 108 | 1.9 |  |  |  |  |  |  |  |  | 91 | 10 | 5 |  | 2 |  |  |  |
|  | *S. warneri* | 33 | 24.2 |  |  |  |  |  |  |  |  | 22 | 2 | 1 | 3 | 5 |  |  |  |
|  | *S. xylosus* | 10 |  |  |  |  |  |  |  |  |  | 10 |  |  |  |  |  |  |  |
|  | *S.* sp | 5 | 20.0 |  |  |  |  |  |  |  |  | 3 | 1 |  |  | 1 |  |  |  |
| Neomycin |  |  |  |  |  |  |  |  |  |  |  | **≤4** | **8** | **16** | **32** | **≥64** |  |  | **1.0^†^** |
|  | *S. agnetis* | 10 |  |  |  |  |  |  |  |  |  | 10 |  |  |  |  |  |  |  |
|  | *S. capitis* | 2 |  |  |  |  |  |  |  |  |  | 2 |  |  |  |  |  |  |  |
|  | *S. chromogenes* | 54 |  |  |  |  |  |  |  |  |  | 54 |  |  |  |  |  |  |  |
|  | *S. cohnii* | 12 |  |  |  |  |  |  |  |  |  | 12 |  |  |  |  |  |  |  |
|  | *S. epidermidis* | 105 |  |  |  |  |  |  |  |  |  | 97 | 3 | 3 | 2 |  |  |  |  |
|  | *S. equorum* | 3 |  |  |  |  |  |  |  |  |  | 3 |  |  |  |  |  |  |  |
|  | *S. haemolyticus* | 42 |  |  |  |  |  |  |  |  |  | 42 |  |  |  |  |  |  |  |
|  | *S. hyicus* | 5 |  |  |  |  |  |  |  |  |  | 5 |  |  |  |  |  |  |  |
|  | *S. kloosii* | 3 |  |  |  |  |  |  |  |  |  | 3 |  |  |  |  |  |  |  |
|  | *S. pasteuri* | 2 |  |  |  |  |  |  |  |  |  | 2 |  |  |  |  |  |  |  |
|  | *S. saprophyticus* | 3 |  |  |  |  |  |  |  |  |  | 3 |  |  |  |  |  |  |  |
|  | *S. sciuri* | 3 |  |  |  |  |  |  |  |  |  | 3 |  |  |  |  |  |  |  |
|  | *S. simulans* | 108 |  |  |  |  |  |  |  |  |  | 108 |  |  |  |  |  |  |  |
|  | *S. warneri* | 33 |  |  |  |  |  |  |  |  |  | 33 |  |  |  |  |  |  |  |
|  | *S. xylosus* | 10 |  |  |  |  |  |  |  |  |  | 10 |  |  |  |  |  |  |  |
|  | *S.* sp | 5 |  |  |  |  |  |  |  |  |  | 5 |  |  |  |  |  |  |  |
| Gentamicin |  |  |  |  |  |  |  |  | **≤0.5** | **1** | **2** | **4** | **8** | **≥16** |  |  |  |  | **0.5** |
|  | *S. agnetis* | 10 |  |  |  |  |  |  | 10 |  |  |  |  |  |  |  |  |  |  |
|  | *S. capitis* | 2 |  |  |  |  |  |  | 2 |  |  |  |  |  |  |  |  |  |  |
|  | *S. chromogenes* | 54 |  |  |  |  |  |  | 54 |  |  |  |  |  |  |  |  |  |  |
|  | *S. cohnii* | 12 |  |  |  |  |  |  | 12 |  |  |  |  |  |  |  |  |  |  |
|  | *S. epidermidis* | 105 |  |  |  |  |  |  | 105 |  |  |  |  |  |  |  |  |  |  |
|  | *S. equorum* | 3 |  |  |  |  |  |  | 3 |  |  |  |  |  |  |  |  |  |  |
|  | *S. haemolyticus* | 42 |  |  |  |  |  |  | 42 |  |  |  |  |  |  |  |  |  |  |
|  | *S. hyicus* | 5 |  |  |  |  |  |  | 5 |  |  |  |  |  |  |  |  |  |  |
|  | *S. kloosii* | 3 |  |  |  |  |  |  | 3 |  |  |  |  |  |  |  |  |  |  |
|  | *S. pasteuri* | 2 |  |  |  |  |  |  | 2 |  |  |  |  |  |  |  |  |  |  |
|  | *S. saprophyticus* | 3 |  |  |  |  |  |  | 3 |  |  |  |  |  |  |  |  |  |  |
|  | *S. sciuri* | 3 |  |  |  |  |  |  | 3 |  |  |  |  |  |  |  |  |  |  |
|  | *S. simulans* | 108 | 2.8 |  |  |  |  |  | 105 | 3 |  |  |  |  |  |  |  |  |  |
|  | *S. warneri* | 33 |  |  |  |  |  |  | 33 |  |  |  |  |  |  |  |  |  |  |
|  | *S. xylosus* | 10 |  |  |  |  |  |  | 10 |  |  |  |  |  |  |  |  |  |  |
|  | *S.* sp | 5 |  |  |  |  |  |  | 5 |  |  |  |  |  |  |  |  |  |  |
| Clindamycin |  |  |  |  |  |  |  |  |  | **≤1** | **2** | **4** | **≥8** |  |  |  |  |  | **0.25** |
|  | *S. agnetis* | 10 |  |  |  |  |  |  |  | 10 |  |  |  |  |  |  |  |  |  |
|  | *S. capitis* | 2 |  |  |  |  |  |  |  | 2 |  |  |  |  |  |  |  |  |  |
|  | *S. chromogenes* | 54 |  |  |  |  |  |  |  | 54 |  |  |  |  |  |  |  |  |  |
|  | *S. cohnii* | 12 |  |  |  |  |  |  |  | 12 |  |  |  |  |  |  |  |  |  |
|  | *S. epidermidis* | 105 |  |  |  |  |  |  |  | 105 |  |  |  |  |  |  |  |  |  |
|  | *S. equorum* | 3 |  |  |  |  |  |  |  | 3 |  |  |  |  |  |  |  |  |  |
|  | *S. haemolyticus* | 42 |  |  |  |  |  |  |  | 41 |  | 1 |  |  |  |  |  |  |  |
|  | *S. hyicus* | 5 |  |  |  |  |  |  |  | 5 |  |  |  |  |  |  |  |  |  |
|  | *S. kloosii* | 3 |  |  |  |  |  |  |  | 3 |  |  |  |  |  |  |  |  |  |
|  | *S. pasteuri* | 2 |  |  |  |  |  |  |  | 2 |  |  |  |  |  |  |  |  |  |
|  | *S. saprophyticus* | 3 |  |  |  |  |  |  |  | 3 |  |  |  |  |  |  |  |  |  |
|  | *S. sciuri* | 3 |  |  |  |  |  |  |  | 3 |  |  |  |  |  |  |  |  |  |
|  | *S. simulans* | 108 |  |  |  |  |  |  |  | 108 |  |  |  |  |  |  |  |  |  |
|  | *S. warneri* | 33 |  |  |  |  |  |  |  | 33 |  |  |  |  |  |  |  |  |  |
|  | *S. xylosus* | 10 |  |  |  |  |  |  |  | 10 |  |  |  |  |  |  |  |  |  |
|  | *S.* sp | 5 |  |  |  |  |  |  |  | 5 |  |  |  |  |  |  |  |  |  |
| Erythromycin |  |  |  |  |  |  |  |  | **≤0.5** | **1** | **2** | **4** | **≥8** |  |  |  |  |  | **1.0** |
|  | *S. agnetis* | 10 |  |  |  |  |  |  | 9 | 1 |  |  |  |  |  |  |  |  |  |
|  | *S. capitis* | 2 |  |  |  |  |  |  | 2 |  |  |  |  |  |  |  |  |  |  |
|  | *S. chromogenes* | 54 | 3.7 |  |  |  |  |  | 51 | 1 |  |  | 2 |  |  |  |  |  |  |
|  | *S. cohnii* | 12 | 58.3 |  |  |  |  |  | 4 | 1 | 2 | 2 | 3 |  |  |  |  |  |  |
|  | *S. epidermidis* | 105 | 11.4 |  |  |  |  |  | 92 | 1 |  |  | 12 |  |  |  |  |  |  |
|  | *S. equorum* | 3 | 33.3 |  |  |  |  |  |  | 2 |  |  | 1 |  |  |  |  |  |  |
|  | *S. haemolyticus* | 42 | 2.4 |  |  |  |  |  | 40 | 1 |  |  | 1 |  |  |  |  |  |  |
|  | *S. hyicus* | 5 |  |  |  |  |  |  | 5 |  |  |  |  |  |  |  |  |  |  |
|  | *S. kloosii* | 3 |  |  |  |  |  |  | 1 | 2 |  |  |  |  |  |  |  |  |  |
|  | *S. pasteuri* | 2 |  |  |  |  |  |  | 2 |  |  |  |  |  |  |  |  |  |  |
|  | *S. saprophyticus* | 3 |  |  |  |  |  |  | 3 |  |  |  |  |  |  |  |  |  |  |
|  | *S. sciuri* | 3 |  |  |  |  |  |  | 3 |  |  |  |  |  |  |  |  |  |  |
|  | *S. simulans* | 108 |  |  |  |  |  |  | 107 | 1 |  |  |  |  |  |  |  |  |  |
|  | *S. warneri* | 33 | 6.1 |  |  |  |  |  | 31 |  | 1 |  | 1 |  |  |  |  |  |  |
|  | *S. xylosus* | 10 |  |  |  |  |  |  | 9 | 1 |  |  |  |  |  |  |  |  |  |
|  | *S.* sp | 5 |  |  |  |  |  |  | 5 |  |  |  |  |  |  |  |  |  |  |
| Chloramphenicol |  |  |  |  |  |  |  |  |  |  | **≤2** | **4** | **8** | **16** | **≥32** |  |  |  | **16.0** |
|  | *S. agnetis* | 10 |  |  |  |  |  |  |  |  |  | 6 | 4 |  |  |  |  |  |  |
|  | *S. capitis* | 2 |  |  |  |  |  |  |  |  |  | 1 | 1 |  |  |  |  |  |  |
|  | *S. chromogenes* | 54 |  |  |  |  |  |  |  |  | 2 | 39 | 13 |  |  |  |  |  |  |
|  | *S. cohnii* | 12 |  |  |  |  |  |  |  |  | 1 | 2 | 9 |  |  |  |  |  |  |
|  | *S. epidermidis* | 105 | 1.9 |  |  |  |  |  |  |  | 1 | 94 | 8 |  | 2 |  |  |  |  |
|  | *S. equorum* | 3 |  |  |  |  |  |  |  |  |  | 1 | 2 |  |  |  |  |  |  |
|  | *S. haemolyticus* | 42 |  |  |  |  |  |  |  |  | 4 | 37 | 1 |  |  |  |  |  |  |
|  | *S. hyicus* | 5 |  |  |  |  |  |  |  |  |  | 4 | 1 |  |  |  |  |  |  |
|  | *S. kloosii* | 3 |  |  |  |  |  |  |  |  |  | 1 | 2 |  |  |  |  |  |  |
|  | *S. pasteuri* | 2 |  |  |  |  |  |  |  |  |  | 4 |  |  |  |  |  |  |  |
|  | *S. saprophyticus* | 3 |  |  |  |  |  |  |  |  |  |  | 3 |  |  |  |  |  |  |
|  | *S. sciuri* | 3 |  |  |  |  |  |  |  |  | 1 | 2 |  |  |  |  |  |  |  |
|  | *S. simulans* | 108 |  |  |  |  |  |  |  |  | 3 | 60 | 43 | 2 |  |  |  |  |  |
|  | *S. warneri* | 33 |  |  |  |  |  |  |  |  | 1 | 22 | 10 |  |  |  |  |  |  |
|  | *S. xylosus* | 10 |  |  |  |  |  |  |  |  |  | 9 | 1 |  |  |  |  |  |  |
|  | *S.* sp | 5 |  |  |  |  |  |  |  |  |  | 5 |  |  |  |  |  |  |  |
| Tetracycline |  |  |  |  |  |  |  |  | **≤0.5** | **1** | **2** | **4** | **8** | **16** | **32** | **64** | **≥128** |  | **1.0** |
|  | *S. agnetis* | 10 |  |  |  |  |  |  | 10 |  |  |  |  |  |  |  |  |  |  |
|  | *S. capitis* | 2 |  |  |  |  |  |  | 2 |  |  |  |  |  |  |  |  |  |  |
|  | *S. chromogenes* | 54 | 5.6 |  |  |  |  |  | 48 | 3 |  |  |  |  |  | 1 | 2 |  |  |
|  | *S. cohnii* | 12 | 16.7 |  |  |  |  |  | 2 | 8 |  |  |  |  |  |  | 2 |  |  |
|  | *S. epidermidis* | 105 | 41.0 |  |  |  |  |  | 55 | 7 | 11 | 16 | 2 |  | 1 | 5 | 8 |  |  |
|  | *S. equorum* | 3 | 66.7 |  |  |  |  |  | 1 |  | 2 |  |  |  |  |  |  |  |  |
|  | *S. haemolyticus* | 42 |  |  |  |  |  |  | 33 | 9 |  |  |  |  |  |  |  |  |  |
|  | *S. hyicus* | 5 |  |  |  |  |  |  | 3 | 2 |  |  |  |  |  |  |  |  |  |
|  | *S. kloosii* | 3 | 33.3 |  |  |  |  |  | 2 |  |  |  |  |  | 1 |  |  |  |  |
|  | *S. pasteuri* | 2 |  |  |  |  |  |  | 1 | 1 |  |  |  |  |  |  |  |  |  |
|  | *S. saprophyticus* | 3 | 33.3 |  |  |  |  |  | 2 |  |  |  |  |  |  |  | 1 |  |  |
|  | *S. sciuri* | 3 |  |  |  |  |  |  | 3 |  |  |  |  |  |  |  |  |  |  |
|  | *S. simulans* | 108 | 5.6 |  |  |  |  |  | 44 | 58 | 2 | 1 |  | 2 |  | 1 |  |  |  |
|  | *S. warneri* | 33 | 27.3 |  |  |  |  |  | 11 | 13 | 1 | 1 |  |  | 1 | 1 | 5 |  |  |
|  | *S. xylosus* | 10 | 10.0 |  |  |  |  |  | 3 | 6 |  |  |  |  |  | 1 |  |  |  |
|  | *S.* sp | 5 |  |  |  |  |  |  | 4 | 1 |  |  |  |  |  |  |  |  |  |
| Trimetoprim- |  |  |  |  |  |  |  |  | **≤0.5/** | **1/** | **2/** | **≥4/** |  |  |  |  |  |  | **0.5/** |
| sulfamethoxazole |  |  |  |  |  |  |  |  | **9.5** | **19** | **38** | **76** |  |  |  |  |  |  | **9.5** |
|  | *S. agnetis* | 10 |  |  |  |  |  |  | 10 |  |  |  |  |  |  |  |  |  |  |
|  | *S. capitis* | 2 |  |  |  |  |  |  | 2 |  |  |  |  |  |  |  |  |  |  |
|  | *S. chromogenes* | 54 | 9.3 |  |  |  |  |  | 49 | 2 | 1 | 2 |  |  |  |  |  |  |  |
|  | *S. cohnii* | 12 |  |  |  |  |  |  | 12 |  |  |  |  |  |  |  |  |  |  |
|  | *S. epidermidis* | 105 | 8.6 |  |  |  |  |  | 96 |  | 4 | 5 |  |  |  |  |  |  |  |
|  | *S. equorum* | 3 |  |  |  |  |  |  | 3 |  |  |  |  |  |  |  |  |  |  |
|  | *S. haemolyticus* | 42 |  |  |  |  |  |  | 42 |  |  |  |  |  |  |  |  |  |  |
|  | *S. hyicus* | 5 | 20.0 |  |  |  |  |  | 4 |  |  | 1 |  |  |  |  |  |  |  |
|  | *S. kloosii* | 3 |  |  |  |  |  |  | 3 |  |  |  |  |  |  |  |  |  |  |
|  | *S. pasteuri* | 2 |  |  |  |  |  |  | 2 |  |  |  |  |  |  |  |  |  |  |
|  | *S. saprophyticus* | 3 |  |  |  |  |  |  | 3 |  |  |  |  |  |  |  |  |  |  |
|  | *S. sciuri* | 3 |  |  |  |  |  |  | 3 |  |  |  |  |  |  |  |  |  |  |
|  | *S. simulans* | 108 | 5.6 |  |  |  |  |  | 102 | 4 | 2 |  |  |  |  |  |  |  |  |
|  | *S. warneri* | 33 |  |  |  |  |  |  | 33 |  |  |  |  |  |  |  |  |  |  |
|  | *S. xylosus* | 10 |  |  |  |  |  |  | 10 |  |  |  |  |  |  |  |  |  |  |
|  | *S.* sp | 5 |  |  |  |  |  |  | 5 |  |  |  |  |  |  |  |  |  |  |
| Trimetoprim |  |  |  |  |  |  |  | **≤0.25** | **0.5** | **1** | **2** | **4** | **8** | **16** | **32** | **≥64** |  |  | **2.0^†^** |
|  | *S. agnetis* | 3 |  |  |  |  |  |  |  |  | 3 |  |  |  |  |  |  |  |  |
|  | *S. capitis* | 1 |  |  |  |  |  |  |  |  | 1 |  |  |  |  |  |  |  |  |
|  | *S. chromogenes* | 6 |  |  |  |  |  |  |  | 3 | 3 |  |  |  |  |  |  |  |  |
|  | *S. cohnii* | 5 | 40.0 |  |  |  |  | 1 |  |  | 2 | 2 |  |  |  |  |  |  |  |
|  | *S. epidermidis* | 27 | 14.8 |  |  |  |  | 3 |  | 12 | 8 |  | 1 | 1 |  | 2 |  |  |  |
|  | *S. equorum* |  |  |  |  |  |  |  |  |  |  |  |  |  |  |  |  |  |  |
|  | *S. haemolyticus* | 5 | 80.0 |  |  |  |  |  |  |  | 1 | 2 |  | 2 |  |  |  |  |  |
|  | *S. hyicus* | 1 | 100.0 |  |  |  |  |  |  |  |  |  |  |  |  | 1 |  |  |  |
|  | *S. kloosii* | 3 |  |  |  |  |  | 1 |  | 2 |  |  |  |  |  |  |  |  |  |
|  | *S. pasteuri* |  |  |  |  |  |  |  |  |  |  |  |  |  |  |  |  |  |  |
|  | *S. saprophyticus* | 1 |  |  |  |  |  | 1 |  |  |  |  |  |  |  |  |  |  |  |
|  | *S. sciuri* | 1 | 100.0 |  |  |  |  |  |  |  |  |  | 1 |  |  |  |  |  |  |
|  | *S. simulans* | 30 | 96.7 |  |  |  |  |  |  |  | 1 | 2 | 11 | 15 | 1 |  |  |  |  |
|  | *S. warneri* | 1 |  |  |  |  |  |  |  | 1 |  |  |  |  |  |  |  |  |  |
|  | *S. xylosus* | 2 | 100.0 |  |  |  |  |  |  |  |  | 1 | 1 |  |  |  |  |  |  |
|  | *S.* sp | 2 | 50.0 |  |  |  |  | 1 |  |  |  |  | 1 |  |  |  |  |  |  |
| Cefoxitin |  |  |  |  |  |  | **≤0.12** | **0.25** | **0.5** | **1** | **2** | **4** | **8** | **16** | **≥32** |  |  |  | **4.0^†^** |
|  | *S. agnetis* | 3 |  |  |  |  |  |  | 3 |  |  |  |  |  |  |  |  |  |  |
|  | *S. capitis* | 1 |  |  |  |  |  |  |  |  |  | 1 |  |  |  |  |  |  |  |
|  | *S. chromogenes* | 6 |  |  |  |  |  |  | 5 | 1 |  |  |  |  |  |  |  |  |  |
|  | *S. cohnii* | 5 | 60.0 |  |  |  |  |  |  |  |  | 2 | 3 |  |  |  |  |  |  |
|  | *S. epidermidis* | 27 | 18.5 |  |  |  |  |  |  | 1 | 15 | 6 | 2 | 1 | 2 |  |  |  |  |
|  | *S. equorum* |  |  |  |  |  |  |  |  |  |  |  |  |  |  |  |  |  |  |
|  | *S. haemolyticus* | 5 |  |  |  |  |  |  |  |  | 2 | 3 |  |  |  |  |  |  |  |
|  | *S. hyicus* | 1 |  |  |  |  |  |  | 1 |  |  |  |  |  |  |  |  |  |  |
|  | *S. kloosii* | 3 | 33.3 |  |  |  |  |  |  |  |  | 2 | 1 |  |  |  |  |  |  |
|  | *S. pasteuri* |  |  |  |  |  |  |  |  |  |  |  |  |  |  |  |  |  |  |
|  | *S. saprophyticus* | 1 |  |  |  |  |  |  |  |  | 1 |  |  |  |  |  |  |  |  |
|  | *S. sciuri* | 1 | 100.0 |  |  |  |  |  |  |  |  |  | 1 |  |  |  |  |  |  |
|  | *S. simulans* | 30 |  |  |  |  |  |  | 1 |  | 24 | 5 |  |  |  |  |  |  |  |
|  | *S. warneri* | 1 |  |  |  |  |  |  |  |  | 1 |  |  |  |  |  |  |  |  |
|  | *S. xylosus* | 2 |  |  |  |  |  |  |  | 1 | 1 |  |  |  |  |  |  |  |  |
|  | *S.* sp | 2 | 50.0 |  |  |  |  |  |  |  | 1 |  | 1 |  |  |  |  |  |  |
| Kanamycin |  |  |  |  |  |  | **≤0.12** | **0.25** | **0.5** | **1** | **2** | **4** | **8** | **16** | **32** | **≥64** |  |  | **8.0^†^** |
|  | *S. agnetis* | 3 |  |  |  |  |  |  |  | 1 | 2 |  |  |  |  |  |  |  |  |
|  | *S. capitis* | 1 |  |  |  |  | 1 |  |  |  |  |  |  |  |  |  |  |  |  |
|  | *S. chromogenes* | 6 |  |  |  |  |  |  | 2 | 4 |  |  |  |  |  |  |  |  |  |
|  | *S. cohnii* | 5 |  |  |  |  | 2 |  | 3 |  |  |  |  |  |  |  |  |  |  |
|  | *S. epidermidis* | 27 | 7.4 |  |  |  |  |  | 5 | 13 | 7 |  |  | 1 |  | 1 |  |  |  |
|  | *S. equorum* |  |  |  |  |  |  |  |  |  |  |  |  |  |  |  |  |  |  |
|  | *S. haemolyticus* | 5 |  |  |  |  |  |  | 4 | 1 |  |  |  |  |  |  |  |  |  |
|  | *S. hyicus* | 1 |  |  |  |  |  |  |  | 1 |  |  |  |  |  |  |  |  |  |
|  | *S. kloosii* | 3 |  |  |  |  |  |  | 3 |  |  |  |  |  |  |  |  |  |  |
|  | *S. pasteuri* |  |  |  |  |  |  |  |  |  |  |  |  |  |  |  |  |  |  |
|  | *S. saprophyticus* | 1 |  |  |  |  | 1 |  |  |  |  |  |  |  |  |  |  |  |  |
|  | *S. sciuri* | 1 |  |  |  |  |  |  |  | 1 |  |  |  |  |  |  |  |  |  |
|  | *S. simulans* | 30 |  |  |  |  |  |  | 9 | 12 | 9 |  |  |  |  |  |  |  |  |
|  | *S. warneri* | 1 |  |  |  |  |  |  |  | 1 |  |  |  |  |  |  |  |  |  |
|  | *S. xylosus* | 2 |  |  |  |  | 2 |  |  |  |  |  |  |  |  |  |  |  |  |
|  | *S.* sp | 2 |  |  |  |  |  |  | 2 |  |  |  |  |  |  |  |  |  |  |
| Florphenicol |  |  |  |  |  |  |  |  |  | **≤1** | **2** | **4** | **8** | **≥16** |  |  |  |  | **8.0^†^** |
|  | *S. agnetis* | 3 |  |  |  |  |  |  |  |  |  | 3 |  |  |  |  |  |  |  |
|  | *S. capitis* | 1 |  |  |  |  |  |  |  |  |  | 1 |  |  |  |  |  |  |  |
|  | *S. chromogenes* | 6 |  |  |  |  |  |  |  | 3 |  | 3 |  |  |  |  |  |  |  |
|  | *S. cohnii* | 5 |  |  |  |  |  |  |  |  |  | 4 | 1 |  |  |  |  |  |  |
|  | *S. epidermidis* | 27 |  |  |  |  |  |  |  | 13 |  | 14 |  |  |  |  |  |  |  |
|  | *S. equorum* |  |  |  |  |  |  |  |  |  |  |  |  |  |  |  |  |  |  |
|  | *S. haemolyticus* | 5 |  |  |  |  |  |  |  | 3 |  | 2 |  |  |  |  |  |  |  |
|  | *S. hyicus* | 1 |  |  |  |  |  |  |  |  |  | 1 |  |  |  |  |  |  |  |
|  | *S. kloosii* | 3 |  |  |  |  |  |  |  | 1 |  | 2 |  |  |  |  |  |  |  |
|  | *S. pasteuri* |  |  |  |  |  |  |  |  |  |  |  |  |  |  |  |  |  |  |
|  | *S. saprophyticus* | 1 |  |  |  |  |  |  |  |  |  | 1 |  |  |  |  |  |  |  |
|  | *S. sciuri* | 1 |  |  |  |  |  |  |  | 1 |  |  |  |  |  |  |  |  |  |
|  | *S. simulans* | 30 |  |  |  |  |  |  |  | 3 |  | 26 | 1 |  |  |  |  |  |  |
|  | *S. warneri* | 1 |  |  |  |  |  |  |  |  |  | 1 |  |  |  |  |  |  |  |
|  | *S. xylosus* | 2 |  |  |  |  |  |  |  |  |  | 2 |  |  |  |  |  |  |  |
|  | *S.* sp | 2 |  |  |  |  |  |  |  |  |  | 2 |  |  |  |  |  |  |  |
| Ciprofloxacin |  |  |  |  |  | **≤0.06** | **0.12** | **0.25** | **0.5** | **≥1** |  |  |  |  |  |  |  |  | **1.0** |
|  | *S. agnetis* | 3 |  |  |  |  | 2 | 1 |  |  |  |  |  |  |  |  |  |  |  |
|  | *S. capitis* | 1 |  |  |  |  |  | 1 |  |  |  |  |  |  |  |  |  |  |  |
|  | *S. chromogenes* | 6 |  |  |  |  | 6 |  |  |  |  |  |  |  |  |  |  |  |  |
|  | *S. cohnii* | 5 |  |  |  |  |  |  | 5 |  |  |  |  |  |  |  |  |  |  |
|  | *S. epidermidis* | 27 |  |  |  |  | 4 | 22 | 1 |  |  |  |  |  |  |  |  |  |  |
|  | *S. equorum* |  |  |  |  |  |  |  |  |  |  |  |  |  |  |  |  |  |  |
|  | *S. haemolyticus* | 5 |  |  |  |  |  | 4 | 1 |  |  |  |  |  |  |  |  |  |  |
|  | *S. hyicus* | 1 |  |  |  |  |  | 1 |  |  |  |  |  |  |  |  |  |  |  |
|  | *S. kloosii* | 3 |  |  |  |  |  |  | 3 |  |  |  |  |  |  |  |  |  |  |
|  | *S. pasteuri* |  |  |  |  |  |  |  |  |  |  |  |  |  |  |  |  |  |  |
|  | *S. saprophyticus* | 1 |  |  |  |  |  |  | 1 |  |  |  |  |  |  |  |  |  |  |
|  | *S. sciuri* | 1 |  |  |  |  |  |  | 1 |  |  |  |  |  |  |  |  |  |  |
|  | *S. simulans* | 30 |  |  |  |  | 15 | 15 |  |  |  |  |  |  |  |  |  |  |  |
|  | *S. warneri* | 1 |  |  |  |  |  | 1 |  |  |  |  |  |  |  |  |  |  |  |
|  | *S. xylosus* | 2 |  |  |  |  |  | 2 |  |  |  |  |  |  |  |  |  |  |  |
|  | *S.* sp | 2 |  |  |  |  | 1 |  | 1 |  |  |  |  |  |  |  |  |  |  |
| Fusidic acid |  |  |  |  |  |  | **≤0.12** | **0.25** | **0.5** | **1** | **2** | **4** | **8** | **≥16** |  |  |  |  | **0.5** |
|  | *S. agnetis* | 3 | 100.0 |  |  |  |  |  |  | 1 | 1 | 1 |  |  |  |  |  |  |  |
|  | *S. capitis* | 1 |  |  |  |  |  |  | 1 |  |  |  |  |  |  |  |  |  |  |
|  | *S. chromogenes* | 6 | 16.7 |  |  |  |  | 4 | 1 | 1 |  |  |  |  |  |  |  |  |  |
|  | *S. cohnii* | 5 | 100.0 |  |  |  |  |  |  |  |  | 3 | 2 |  |  |  |  |  |  |
|  | *S. epidermidis* | 27 | 37.0 |  |  |  |  | 1 | 16 | 7 |  |  | 1 | 2 |  |  |  |  |  |
|  | *S. equorum* |  |  |  |  |  |  |  |  |  |  |  |  |  |  |  |  |  |  |
|  | *S. haemolyticus* | 5 |  |  |  |  |  |  | 5 |  |  |  |  |  |  |  |  |  |  |
|  | *S. hyicus* | 1 |  |  |  |  |  |  | 1 |  |  |  |  |  |  |  |  |  |  |
|  | *S. kloosii* | 3 | 100.0 |  |  |  |  |  |  | 1 | 1 | 1 |  |  |  |  |  |  |  |
|  | *S. pasteuri* |  |  |  |  |  |  |  |  |  |  |  |  |  |  |  |  |  |  |
|  | *S. saprophyticus* | 1 | 100.0 |  |  |  |  |  |  |  | 1 |  |  |  |  |  |  |  |  |
|  | *S. sciuri* | 1 | 100.0 |  |  |  |  |  |  |  |  | 1 |  |  |  |  |  |  |  |
|  | *S. simulans* | 30 | 33.3 |  |  |  |  | 2 | 18 | 10 |  |  |  |  |  |  |  |  |  |
|  | *S. warneri* | 1 | 100.0 |  |  |  |  |  |  | 1 |  |  |  |  |  |  |  |  |  |
|  | *S. xylosus* | 2 | 100.0 |  |  |  |  |  |  | 1 | 1 |  |  |  |  |  |  |  |  |
|  | *S.* sp | 2 | 50.0 |  |  |  |  | 1 |  |  |  | 1 |  |  |  |  |  |  |  |
| Vancomycin |  |  |  |  |  |  |  |  |  | **≤1** | **2** | **4** | **8** | **16** | **32** | **≥64** |  |  | **4.0** |
|  | *S. agnetis* | 7 |  |  |  |  |  |  |  | 7 |  |  |  |  |  |  |  |  |  |
|  | *S. capitis* | 1 |  |  |  |  |  |  |  | 1 |  |  |  |  |  |  |  |  |  |
|  | *S. chromogenes* | 48 |  |  |  |  |  |  |  | 40 | 8 |  |  |  |  |  |  |  |  |
|  | *S. cohnii* | 7 |  |  |  |  |  |  |  | 4 | 3 |  |  |  |  |  |  |  |  |
|  | *S. epidermidis* | 78 |  |  |  |  |  |  |  | 21 | 57 |  |  |  |  |  |  |  |  |
|  | *S. equorum* | 3 |  |  |  |  |  |  |  | 1 | 2 |  |  |  |  |  |  |  |  |
|  | *S. haemolyticus* | 37 |  |  |  |  |  |  |  | 30 | 7 |  |  |  |  |  |  |  |  |
|  | *S. hyicus* | 4 |  |  |  |  |  |  |  | 3 | 1 |  |  |  |  |  |  |  |  |
|  | *S. kloosii* |  |  |  |  |  |  |  |  |  |  |  |  |  |  |  |  |  |  |
|  | *S. pasteuri* | 2 |  |  |  |  |  |  |  | 1 | 1 |  |  |  |  |  |  |  |  |
|  | *S. saprophyticus* | 2 |  |  |  |  |  |  |  | 2 |  |  |  |  |  |  |  |  |  |
|  | *S. sciuri* | 2 |  |  |  |  |  |  |  | 2 |  |  |  |  |  |  |  |  |  |
|  | *S. simulans* | 78 | 1.3 |  |  |  |  |  |  | 71 | 5 | 1 |  |  | 1 |  |  |  |  |
|  | *S. warneri* | 32 |  |  |  |  |  |  |  | 27 | 5 |  |  |  |  |  |  |  |  |
|  | *S. xylosus* | 8 |  |  |  |  |  |  |  | 5 | 3 |  |  |  |  |  |  |  |  |
|  | *S.* sp | 3 |  |  |  |  |  |  |  | 2 | 1 |  |  |  |  |  |  |  |  |
| Virginiamycin |  |  |  |  |  |  |  |  | **≤0.5** | **1** | **2** | **4** | **8** | **16** | **32** | **≥64** |  |  | **1.0^‡^** |
|  | *S. agnetis* | 7 |  |  |  |  |  |  | 5 | 2 |  |  |  |  |  |  |  |  |  |
|  | *S. capitis* | 1 |  |  |  |  |  |  |  | 1 |  |  |  |  |  |  |  |  |  |
|  | *S. chromogenes* | 48 | 4.2 |  |  |  |  |  | 12 | 34 | 2 |  |  |  |  |  |  |  |  |
|  | *S. cohnii* | 7 | 71.4 |  |  |  |  |  | 1 | 1 | 5 |  |  |  |  |  |  |  |  |
|  | *S. epidermidis* | 78 | 1.3 |  |  |  |  |  | 51 | 26 | 1 |  |  |  |  |  |  |  |  |
|  | *S. equorum* | 3 | 66.7 |  |  |  |  |  |  | 1 | 2 |  |  |  |  |  |  |  |  |
|  | *S. haemolyticus* | 37 | 2.7 |  |  |  |  |  | 14 | 22 | 1 |  |  |  |  |  |  |  |  |
|  | *S. hyicus* | 4 |  |  |  |  |  |  | 1 | 3 |  |  |  |  |  |  |  |  |  |
|  | *S. kloosii* |  |  |  |  |  |  |  |  |  |  |  |  |  |  |  |  |  |  |
|  | *S. pasteuri* | 2 |  |  |  |  |  |  | 1 | 1 |  |  |  |  |  |  |  |  |  |
|  | *S. saprophyticus* | 2 | 100.0 |  |  |  |  |  |  |  | 2 |  |  |  |  |  |  |  |  |
|  | *S. sciuri* | 2 | 100.0 |  |  |  |  |  |  |  | 2 |  |  |  |  |  |  |  |  |
|  | *S. simulans* | 78 | 2.6 |  |  |  |  |  | 12 | 64 | 1 | 1 |  |  |  |  |  |  |  |
|  | *S. warneri* | 32 | 15.6 |  |  |  |  |  | 6 | 21 | 4 | 1 |  |  |  |  |  |  |  |
|  | *S. xylosus* | 8 | 75.0 |  |  |  |  |  |  | 2 | 3 | 3 |  |  |  |  |  |  |  |
|  | *S.* sp | 3 | 33.3 |  |  |  |  |  | 1 | 1 |  | 1 |  |  |  |  |  |  |  |
| Avilamycin |  |  |  |  |  |  |  |  | **≤0.5** | **1** | **2** | **4** | **8** | **16** | **32** | **≥64** |  |  | **ND** |
|  | *S. agnetis* | 7 |  |  |  |  |  |  |  |  |  | 5 | 2 |  |  |  |  |  |  |
|  | *S. capitis* | 1 |  |  |  |  |  |  |  |  |  |  | 1 |  |  |  |  |  |  |
|  | *S. chromogenes* | 48 |  |  |  |  |  |  |  |  | 13 | 26 | 9 |  |  |  |  |  |  |
|  | *S. cohnii* | 7 |  |  |  |  |  |  |  |  |  |  | 2 | 3 | 1 | 1 |  |  |  |
|  | *S. epidermidis* | 78 |  |  |  |  |  |  |  |  |  | 23 | 51 | 4 |  |  |  |  |  |
|  | *S. equorum* | 3 |  |  |  |  |  |  |  |  |  | 1 |  | 2 |  |  |  |  |  |
|  | *S. haemolyticus* | 37 |  |  |  |  |  |  |  |  | 6 | 22 | 8 | 1 |  |  |  |  |  |
|  | *S. hyicus* | 4 |  |  |  |  |  |  |  |  |  | 2 | 2 |  |  |  |  |  |  |
|  | *S. kloosii* |  |  |  |  |  |  |  |  |  |  |  |  |  |  |  |  |  |  |
|  | *S. pasteuri* | 2 |  |  |  |  |  |  |  |  |  |  | 2 |  |  |  |  |  |  |
|  | *S. saprophyticus* | 2 |  |  |  |  |  |  |  |  |  | 1 |  | 1 |  |  |  |  |  |
|  | *S. sciuri* | 2 |  |  |  |  |  |  |  |  | 1 | 1 |  |  |  |  |  |  |  |
|  | *S. simulans* | 78 |  |  |  |  |  |  |  | 1 | 1 | 9 | 29 | 26 | 12 |  |  |  |  |
|  | *S. warneri* | 32 |  |  |  |  |  |  |  |  |  | 7 | 23 | 2 |  |  |  |  |  |
|  | *S. xylosus* | 8 |  |  |  |  |  |  |  |  |  | 2 | 6 |  |  |  |  |  |  |
|  | *S.* sp | 3 |  |  |  |  |  |  |  |  |  | 2 |  | 1 |  |  |  |  |  |

*Current (March 2015) EUCAST epidemiological cut-off (ECOFF) values (mg/l) for CNS were used to define resistant isolates. If ECOFF for CNS was not available, the value for *S. aureus* or *S. intermedius* was used.

^†^ECOFF for *S. aureus*

^‡^ECOFF for *S. intermedius*

ND = not determined
