# Supplementary material for: Species distribution and in vitro antimicrobial susceptibility of coagulase-negative staphylococci isolated from bovine mastitic milk
Source: Acta Vet Scand. 2016 Feb 6;58:12. doi: 10.1186/s13028-016-0193-8 (PMC4744398; doi:10.1186/s13028-016-0193-8)
Supplement: Supplementary file 2 — Additional file 2: Table S2. In vitro susceptibility to 20 antimicrobials of coagulase-negative Staphylococcus isolates from bovine milk samples from dataset 1 (2001, 312 isolates) [2] and 2 (2012, 88 isolates) [18]. [file 13028_2016_193_MOESM2_ESM.docx]

Table S2. *In vitro* susceptibility to 20 antimicrobials of coagulase-negative *Staphylococcus* isolates from bovine milk samples from dataset 1 (2001, 312 isolates) [2] and 2 (2012, 88 isolates) [18].

|  | **Year** | **N** | **% >ECOFF** | Number of isolates with indicated MIC, mg/l | | | | | | | | | | | | | | | **ECOFF*** |
| --- | --- | --- | --- | --- | --- | --- | --- | --- | --- | --- | --- | --- | --- | --- | --- | --- | --- | --- | --- |
| **Benzylpenicillin** |  |  |  |  |  | **≤0.06** | **0.12** | **0.25** | **0.5** | **1** | **2** | **4** | **≥8** |  |  |  |  |  | **0.125^†^** |
|  | 2001 | 312 | 41.7 |  |  | 160 | 22 | 25 | 28 | 26 | 24 | 9 | 18 |  |  |  |  |  |  |
|  | 2012 | 88 | 39.8 |  |  | 49 | 4 | 12 | 3 | 9 | 3 | 6 | 2 |  |  |  |  |  |  |
|  | **Total** | **400** | **41.8** |  |  | **209** | **24** | **38** | **32** | **35** | **27** | **15** | **20** |  |  |  |  |  |  |
| **Oxacillin** |  |  |  |  |  |  |  |  | **≤0.5** | **1** | **2** | **4** | **≥8** |  |  |  |  |  | **1.0** |
|  | 2001 | 312 | 37.5 |  |  |  |  |  | 87 | 108 | 87 | 19 | 11 |  |  |  |  |  |  |
|  | 2012 | 88 | 21.6 |  |  |  |  |  | 46 | 23 | 7 | 7 | 5 |  |  |  |  |  |  |
|  | **Total** | **400** | **34.0** |  |  |  |  |  | **133** | **131** | **94** | **26** | **16** |  |  |  |  |  |  |
| **Cephalothin** |  |  |  |  |  |  | **≤0.12** | **0.25** | **0.5** | **1** | **2** | **4** | **≥8** |  |  |  |  |  | **1.0^†^** |
|  | 2001 | 312 | 1.0 |  |  |  | 41 | 183 | 64 | 21 | 3 |  |  |  |  |  |  |  |  |
|  | 2012 | 88 | 0.0 |  |  |  | 20 | 44 | 15 | 9 |  |  |  |  |  |  |  |  |  |
|  | **Total** | **400** | **0.8** |  |  |  | **61** | **227** | **79** | **30** | **3** |  |  |  |  |  |  |  |  |
| **Streptomycin** |  |  |  |  |  |  |  |  |  |  |  | **≤4** | **8** | **16** | **32** | **≥64** |  |  | **16.0^†^** |
|  | 2001 | 312 | 8.3 |  |  |  |  |  |  |  |  | 267 | 12 | 7 | 5 | 21 |  |  |  |
|  | 2012 | 88 | 4.5 |  |  |  |  |  |  |  |  | 70 | 12 | 2 | 1 | 3 |  |  |  |
|  | **Total** | **400** | **7.5** |  |  |  |  |  |  |  |  | **337** | **24** | **9** | **6** | **24** |  |  |  |
| **Neomycin** |  |  |  |  |  |  |  |  |  |  |  | **≤4** | **8** | **16** | **32** | **≥64** |  |  | **1.0^†^** |
|  | 2001 | 312 |  |  |  |  |  |  |  |  |  | 305 | 3 | 3 | 1 |  |  |  |  |
|  | 2012 | 88 |  |  |  |  |  |  |  |  |  | 87 |  |  | 1 |  |  |  |  |
|  | **Total** | **400** |  |  |  |  |  |  |  |  |  | **392** | **3** | **3** | **2** |  |  |  |  |
| **Gentamicin** |  |  |  |  |  |  |  |  | **≤0.5** | **1** | **2** | **4** | **8** | **≥16** |  |  |  |  | **0.5** |
|  | 2001 | 312 | 0.6 |  |  |  |  |  | 310 | 2 |  |  |  |  |  |  |  |  |  |
|  | 2012 | 88 | 1.1 |  |  |  |  |  | 87 | 1 |  |  |  |  |  |  |  |  |  |
|  | **Total** |  | **0.8** |  |  |  |  |  | **397** | **3** |  |  |  |  |  |  |  |  |  |
| **Clindamycin** |  |  |  |  |  |  |  |  |  | **≤1** | **2** | **4** | **≥8** |  |  |  |  |  | **0.25** |
|  | 2001 | 312 |  |  |  |  |  |  |  | 311 |  | 1 |  |  |  |  |  |  |  |
|  | 2012 | 88 |  |  |  |  |  |  |  | 88 |  |  |  |  |  |  |  |  |  |
|  | **Total** | **400** |  |  |  |  |  |  |  | **399** |  | **1** |  |  |  |  |  |  |  |
| **Erythromycin** |  |  |  |  |  |  |  |  | **≤0.5** | **1** | **2** | **4** | **≥8** |  |  |  |  |  | **1.0** |
|  | 2001 | 312 | 6.4 |  |  |  |  |  | 286 | 6 | 2 | 1 | 17 |  |  |  |  |  |  |
|  | 2012 | 88 | 5.7 |  |  |  |  |  | 78 | 5 | 1 | 1 | 3 |  |  |  |  |  |  |
|  | **Total** | **400** | **6.3** |  |  |  |  |  | **364** | **11** | **3** | **2** | **20** |  |  |  |  |  |  |
| **Chloramphenicol** |  |  |  |  |  |  |  |  |  |  | **≤2** | **4** | **8** | **16** | **≥32** |  |  |  | **16.0** |
|  | 2001 | 312 | 0.6 |  |  |  |  |  |  |  | 12 | 220 | 76 | 2 | 2 |  |  |  |  |
|  | 2012 | 88 | 0.0 |  |  |  |  |  |  |  | 1 | 64 | 23 |  |  |  |  |  |  |
|  | **Total** | **400** | **0.5** |  |  |  |  |  |  |  | **13** | **284** | **99** | **2** | **2** |  |  |  |  |
| **Tetracycline** |  |  |  |  |  |  |  |  | **≤0.5** | **1** | **2** | **4** | **8** | **16** | **32** | **64** | **≥128** |  | **1.0** |
|  | 2001 | 312 | 17.9 |  |  |  |  |  | 152 | 104 | 9 | 18 | 2 |  | 2 | 7 | 18 |  |  |
|  | 2012 | 88 | 11.4 |  |  |  |  |  | 72 | 6 | 5 |  |  | 2 | 1 | 2 |  |  |  |
|  | **Total** | **400** | **16.5** |  |  |  |  |  | **224** | **110** | **14** | **18** | **2** | **2** | **3** | **9** | **18** |  |  |
| **Trimetoprim-** |  |  |  |  |  |  |  |  | **≤0.5/** | **1/** | **2/** | **≥4/** |  |  |  |  |  |  | **0.5/** |
| **sulfamethoxazole** |  |  |  |  |  |  |  |  | **9.5** | **19** | **38** | **76** |  |  |  |  |  |  | **9.5** |
|  | 2001 | 312 | 5.8 |  |  |  |  |  | 294 | 6 | 6 | 6 |  |  |  |  |  |  |  |
|  | 2012 | 88 | 3.4 |  |  |  |  |  | 85 |  |  | 3 |  |  |  |  |  |  |  |
|  | **Total** | **400** | **5.3** |  |  |  |  |  | **379** | **6** | **6** | **9** |  |  |  |  |  |  |  |
| **Vancomycin** |  |  |  |  |  |  |  |  |  | **≤1** | **2** | **4** | **8** | **16** | **32** | **≥64** |  |  | **4.0** |
|  | 2001 | 312 | 0.3 |  |  |  |  |  |  | 217 | 93 | 1 |  |  | 1 |  |  |  |  |
|  | **Total** | **312** | **0.3** |  |  |  |  |  |  | **217** | **93** | **1** |  |  | **1** |  |  |  |  |
| **Virginiamycin** |  |  |  |  |  |  |  |  | **≤0.5** | **1** | **2** | **4** | **8** | **16** | **32** | **≥64** |  |  | **1.0^‡^** |
|  | 2001 | 312 | 9.3 |  |  |  |  |  | 104 | 179 | 23 | 6 |  |  |  |  |  |  |  |
|  | **Total** | **312** | **9.3** |  |  |  |  |  | **104** | **179** | **23** | **6** |  |  |  |  |  |  |  |
| **Avilamycin** |  |  |  |  |  |  |  |  | **≤0.5** | **1** | **2** | **4** | **8** | **16** | **32** | **≥64** |  |  | **ND** |
|  | 2001 | 312 |  |  |  |  |  |  |  | 1 | 21 | 101 | 135 | 40 | 13 | 1 |  |  |  |
|  | **Total** | **312** |  |  |  |  |  |  |  | **1** | **21** | **101** | **135** | **40** | **13** | **1** |  |  |  |
| **Trimetoprim** |  |  |  |  |  |  |  |  | **≤0.5** | **1** | **2** | **4** | **8** | **16** | **32** | **≥64** |  |  | **2.0^†^** |
|  | 2012 | 88 | 50.0 |  |  |  |  |  | 7 | 18 | 19 | 7 | 15 | 18 | 1 | 3 |  |  |  |
|  | **Total** | **88** | **50.0** |  |  |  |  |  | **7** | **18** | **19** | **7** | **15** | **18** | **1** | **3** |  |  |  |
| **Cefoxitin** |  |  |  |  |  |  | **≤0.12** | **0.25** | **0.5** | **1** | **2** | **4** | **8** | **16** | **≥32** |  |  |  | **4.0^†^** |
|  | 2012 | 88 | 12.5 |  |  |  |  |  | 10 | 3 | 45 | 19 | 8 | 1 | 2 |  |  |  |  |
|  | **Total** | **88** | **12.5** |  |  |  |  |  | **10** | **3** | **45** | **19** | **8** | **1** | **2** |  |  |  |  |
| **Kanamycin** |  |  |  |  |  |  | **≤0.12** | **0.25** | **0.5** | **1** | **2** | **4** | **8** | **16** | **32** | **≥64** |  |  | **8.0^†^** |
|  | 2012 | 88 | 2.3 |  |  |  | 6 |  | 28 | 34 | 18 |  |  | 1 |  | 1 |  |  |  |
|  | **Total** | **88** | **2.3** |  |  |  | **6** |  | **28** | **34** | **18** |  |  | **1** |  | **1** |  |  |  |
| **Florphenicol** |  |  |  |  |  |  |  |  |  |  | **≤2** | **4** | **8** | **≥16** |  |  |  |  | **8.0^†^** |
|  | 2012 | 88 | 0.0 |  |  |  |  |  |  |  | 24 | 62 | 2 |  |  |  |  |  |  |
|  | **Total** | **88** | **0.0** |  |  |  |  |  |  |  | **24** | **62** | **2** |  |  |  |  |  |  |
| **Ciprofloxacin** |  |  |  |  |  | **≤0.06** | **0.12** | **0.25** | **0.5** | **≥1** |  |  |  |  |  |  |  |  | **1.0** |
|  | 2012 | 88 | 0.0 |  |  |  | 28 | 47 | 13 |  |  |  |  |  |  |  |  |  |  |
|  | **Total** | **88** | **0.0** |  |  |  | **28** | **47** | **13** |  |  |  |  |  |  |  |  |  |  |
| **Fusidic acid** |  |  |  |  |  | **≤0.06** | **0.12** | **0.25** | **0.5** | **1** | **2** | **4** | **8** | **≥16** |  |  |  |  | **0.5** |
|  | 2012 | 88 | 43.2 |  |  |  |  | 8 | 42 | 22 | 4 | 7 | 3 | 2 |  |  |  |  |  |
|  | **Total** | **88** | **43.2** |  |  |  |  | **8** | **42** | **22** | **4** | **7** | **3** | **2** |  |  |  |  |  |

*Current (March 2015) EUCAST epidemiological cut-off (ECOFF) values (mg/l) for CNS were used to define resistant isolates. If ECOFF for CNS was not available, the value for *S. aureus* or *S. intermedius* was used.

^†^ECOFF for *S. aureus*

^‡^ECOFF for *S. intermedius*

ND = not determined
